# Bathyptilones: Terpenoids from an Antarctic Sea Pen, *Anthoptilum grandiflorum* (Verrill, 1879)

**DOI:** 10.3390/md17090513

**Published:** 2019-09-01

**Authors:** Santana A.L. Thomas, Anthony Sanchez, Younghoon Kee, Nerida G. Wilson, Bill J. Baker

**Affiliations:** 1 Department of Chemistry, University of South Florida, Tampa, FL 33620, USA; 2 Department of Cellular, Microbiology and Molecular Biology, University of South Florida, Tampa, FL 33620, USA; 3 Western Australia Museum and the University of Western Australia, Perth, WA 6106, Australia

**Keywords:** briarane diterpene, cytotoxicity screening, deep sea, pennatulacea, marine natural products

## Abstract

An Antarctic coral belonging to the order Pennatulacea, collected during the 2013 austral autumn by trawl from 662 to 944 m depth, has yielded three new briarane diterpenes, bathyptilone A-C (**1**–**3**) along with a trinorditerpene, enbepeanone A (**4**), which bears a new carbon skeleton. Structure elucidation was facilitated by one- and two-dimensional NMR spectroscopy, mass spectrometry and confirmed by X-ray crystallography. The three compounds were screened in four cancer cell lines. Bathyptilone A displayed selective nanomolar cytotoxicity against the neurogenic mammalian cell line Ntera-2.

## 1. Introduction

Since the late 1950s, marine organisms have been increasingly important as sources of novel secondary metabolites, many of which demonstrate significant biological activity. Chemical studies were enabled by technological advancements such as the development of SCUBA diving equipment in the 1950s and 1960s, and of ROVs toward the latter part of the century, providing access to increasingly diverse biological materials. However, marine organisms found in cold-water environments are still largely understudied as sources of new chemodiversity [1]. More than 90% of the ocean exists at temperatures below 3 °C, yet only 2% of natural products are reported from marine organisms collected in these regions [1,2], reflecting, in part, that such environments remain difficult to access. Despite the lack of attention, important biologically active compounds continue to be found [3,4,5,6,7,8,9]. 

**Scheme 1:**
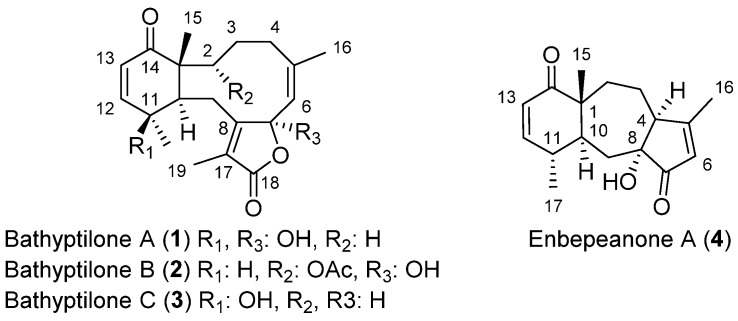
Structure and numbering system for bathyptilones A-C and enbepeanone A.

Species from the order Pennatulacea are known for the production of briarane, cembrane and similar diterpenes [10]. Briarane diterpenes were first described four decades ago and the ensuing years have seen more than 600 reported in the literature [11]. They exhibited a wide range of biological activities, including cytotoxicity, anti-inflammatory, antiviral, insecticidal and immunomodulatory activity [12,13,14,15,16]. A benthic collection of the Pennatulacean genus *Anthoptilum* from Western Australia produced five neuroactive briaranes, the anthoptilides [17]. Herein, we describe the isolation and structure elucidation of two classes of compounds isolated from an Antarctic specimen of *Anthoptilum grandiflorum*, including (Scheme 1) briaranes, bathyptilone A–C (**1**–**3**) and the new scaffold of a trinorditerpene, enbepeanone A (**4**), likely derived from a briarane precursor. 

## 2. Results and Discussion

The coral *Anthoptilum grandiflorum* was collected by trawl at a depth of between 662 and 944 m during an austral autumn cruise aboard the R/V Nathaniel B. Palmer (NBP) in the vicinity of the Scotia Arc. The sea pens were frozen and freeze-dried, followed by an exhaustive extraction in refluxing DCM using a Soxhlet extractor. Normal-phase MPLC of the 10.6 g Soxhlet extract was conducted for initial fractionation. Normal- and reverse-phase HPLC yielded four new terpenoid metabolites (**1**–**4**). 

Bathyptilone A (**1**) was isolated as a crystalline solid with a molecular formula of C_20_H_26_O_5_, based on HRESIMS with supporting ^1^H and ^13^C NMR data (Table 1). The ^13^C NMR spectrum indicated the presence of a ketone, an ester-equivalent, four quaternary, four methine, four methylene and four methyl groups, accounting for all carbons and twenty-four of the twenty-six protons. Further, one quaternary carbon had a shift (δ_c_ 69.3) indicative of an oxygen-bearing sp^3^ carbon, while a second quaternary carbon (δ_c_ 106.2) appeared as a ketal-type carbon. A tricyclic scaffold was derived by the degree of unsaturation, as five of the eight unsaturations could be assigned to double bond functions (ketone, δ_C_ 203.7; ester-equivalent, δ_C_ 171.7; and six olefinic carbons, δ_C_ 160.7, 151.7, 145.3, 127.4, 125.8, 124.1). 

**Figure 1:**
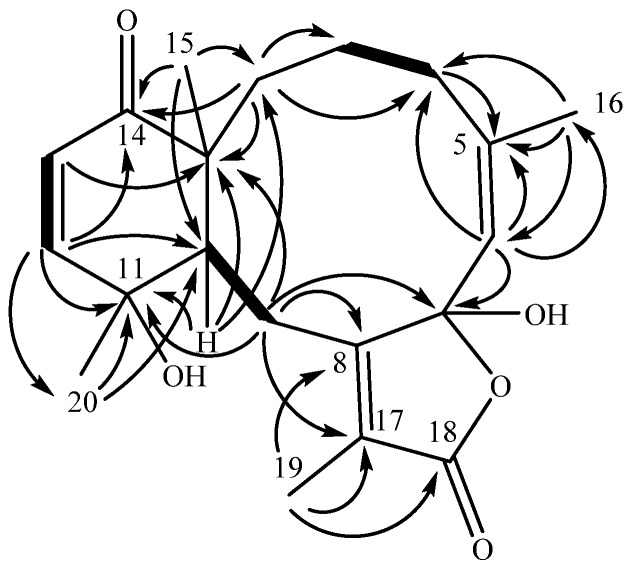
Key COSY (**▬**) and HMBC (→) correlations establishing the planar structure of bathyptilone A (**1**).

A cyclohexenone ring was constructed from COSY and HMBC data. Two ^3^*J*_HH_ coupled olefinic protons (Figure 1), δ_H_ 6.63 (H-12) and δ_H_ 5.97 (H-13), displayed HMBC correlations to δ_C_ 203.7 (C-14), establishing an α,β-unsaturated ketone. Proton H-12 displayed further HMBC connectivity to an aliphatic methine δ_C_ 41.4 (C-10), as well as to a quaternary, oxygen-bearing carbon (δ_C_ 69.3, C-11). Proton H-13 similarly extended the cyclohexenone substructure with HMBC correlation to an aliphatic quaternary carbon (δ_C_ 50.2, C-1), which, by virtue of correlations from a methyl singlet that networked C-1, -10, -14, into the HMBC spin system, completed the cyclohexenone substructure (Figure 1). The cyclohexenone portion could be decorated at C-11 with the C-20 methyl group (δ_C_ 30.4), which displayed HMBC correlations to C-10, -11 and -12 (δ_C_ 151.7). Additional corroboration of the six-membered ring was obtained from H-10, with HMBC correlations to C-1 and C-11. This cyclohexenone partial structure has two open valences, at C-1 and C-10.

The elaboration of the cyclohexenone resulted in a fused cyclodecene ring. Besides previously described correlations into the cyclohexenone ring, H_3_-15 (δ_H_ 1.16) displayed a further correlation to a methylene carbon at δ_C_ 21.8 (C-2). Although proton H-2a (δ_H_ 1.54) and H-2b (δ_H_ 1.84) overlapped protons of H-3a (δ_H_ 1.52) and H-4a (δ_H_ 1.85), respectively, preventing clear assignment of COSY correlations, it was observed that H-2b and C-4 (δ_C_ 29.5) were correlated in the HMBC spectrum. Both H-3a and H-3b displayed HMBC correlations to C-2, and H-3b further correlated to C-1 (δ_C_ 50.2), establishing an alkyl substituent at C-1. Proton H-4a was found to extend the alkyl group through HMBC correlations to two olefinic carbons, C-5 (δ_C_ 145.3) and C-6 (δ_C_ 124.1). The HMBC also established that quaternary C-5 bears a vinyl methyl δ_H_ 1.78 (H_3_-16) based on HMBC correlations of H-6 (δ_C_ 5.35) to C-16 (δ_C_ 23.5) and reciprocal correlations from H_3_-16 to C-6, as well as C-5 (δ_C_ 145.3). Further development of this partial structure ended at a quaternary, acetal-type carbon at δ_C_ 106.2 (C-7) based on an HMBC correlation from H-6.

An isolated spin system was observed in the COSY spectrum between the broad doublet δ_H_ 3.14 (H-10) and the doublet of doublets δ_H_ 3.25 (H-9b), the latter of which also correlated to H-9a (δ_H_ 2.47). HMBC extended that system further based on H_2_-9 correlations to two quaternary olefinic carbons, C-8 (δ_C_ 160.7) and C-17 (δ_C_ 125.8). A key correlation between H-9a and the acetal-like carbon C-7 (δc 106.2) completed a C_10_ macrocycle. 

Remaining to be accounted were an ester-type carbon (δ_C_ 171.7, C-18) and a vinyl methyl group (δ_H_ 1.95, H_3_-19), and two open valencies on C-7 (δc 106.2) and C-17 (δ_C_ 125.8). The vinyl methyl group correlated in the HMBC spectrum to quaternary olefins C-8 and C-17, locating the methyl group on one of the open valencies on C-17. Methyl protons H_3_-19 also displayed an HMBC correlation to C-18, accounting for all the carbon atoms in the molecular formula and requiring that the remaining two oxygen atoms occupy the open valencies on C-7 and C-18, resulting in a γ-lactone bearing a C-7 hemiacetal. The planar structure that results from these connections identifies bathyptilone A (**1**) as a member of the briarane family of octocoral metabolites.

**Figure 2:**
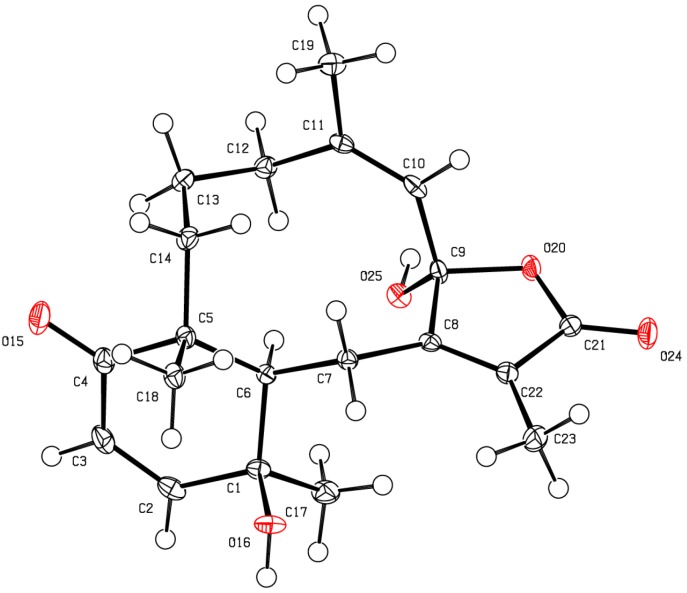
Asymmetric unit of bathyptilone A (**1**). Anisotropic displacement parameters were drawn at 50% probability.

The stereochemical evaluation of bathyptilone A (**1**) was approached using NOESY spectroscopy. Proton H-10 (δ_H_ 3.14) showed NOESY correlations to δ_H_ 1.35 (H_3_-20) and δ_H_ 3.52 (H-4b), while H_2_-9 (δ_H_ 2.47, H-9a; 3.25, H-9b) displayed proximity to H_3_-15 (δ_H_ 1.16), setting the ring fusion *trans*, as observed in all other briarane diterpenes [11], with H_3_-20 occupying the same face of the ring fusion as H-10. The remaining stereogenic carbon, C-7, lacked a proton, but correlation between the 7-OH/11-OH overlapping hydroxyl signal in the ^1^H NMR spectrum, δ_H_ 3.52, and H-10 must result from 7-OH rather than 11-OH, which has already been shown to be on the opposite face of the tricycle from H-10. These stereocenters, as well as the configuration of the C-7 hemiacetal, were confirmed by X-ray crystallography analysis (Figure 2, Table S1), which facilitated the assignment of the absolute configuration of bathyptilone A as depicted in **1**.

**Figure 3:**
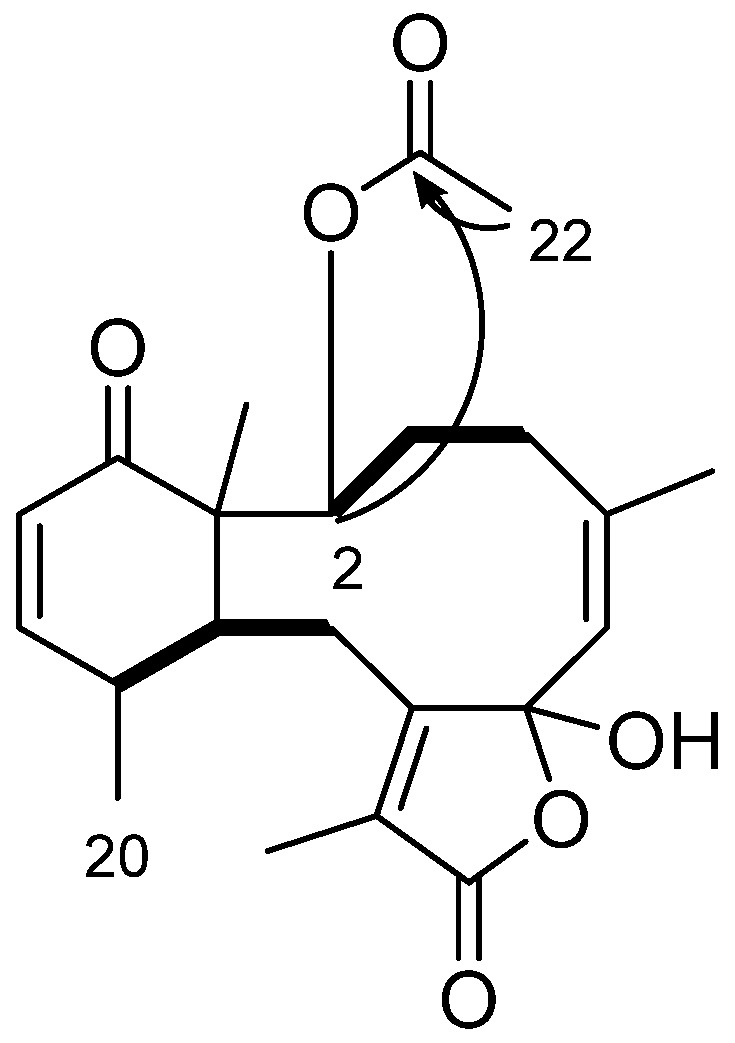
Key COSY (**▬**) and HMBC (→) correlations establishing the differences between bathyptilone A (**1**) and B (**2**).

The molecular formula of bathyptilone B (**2**) was established as C_22_H_28_O_6_ by HRESIMS and supported by ^1^H and ^13^C NMR data (Table 2), which appears to differ by the acetylation of bathyptilone A (**1**). This was confirmed by observation of ^13^C NMR signals for the two additional carbons as an ester-type carbonyl at δ_C_ 169.5 (C-21) and an aliphatic methyl at δ_C_ 21.2 (C-22), the latter bearing protons that were found in the ^1^H NMR spectrum as a 3H singlet at δ_H_ 2.03 (H_3_-22). This substituent could be placed at C-2 (δ_C_ 77.9) based on a deshielded proton at δ_H_ 5.89 (H-2), which correlated in the HMBC spectrum to carbons that matched their bathyptilone A analog, including δ_C_ 52.5 (C-1) and δ_C_ 41.4 (C-10), as well as the new carbon at C-21 (Figure 3). With new oxidation at C-2, relative to bathyptilone A, C-11 (δ_C_ 38.0) was compared between **1** and **2** and found to be proton bearing rather than oxygen bearing. 

**Figure 4:**
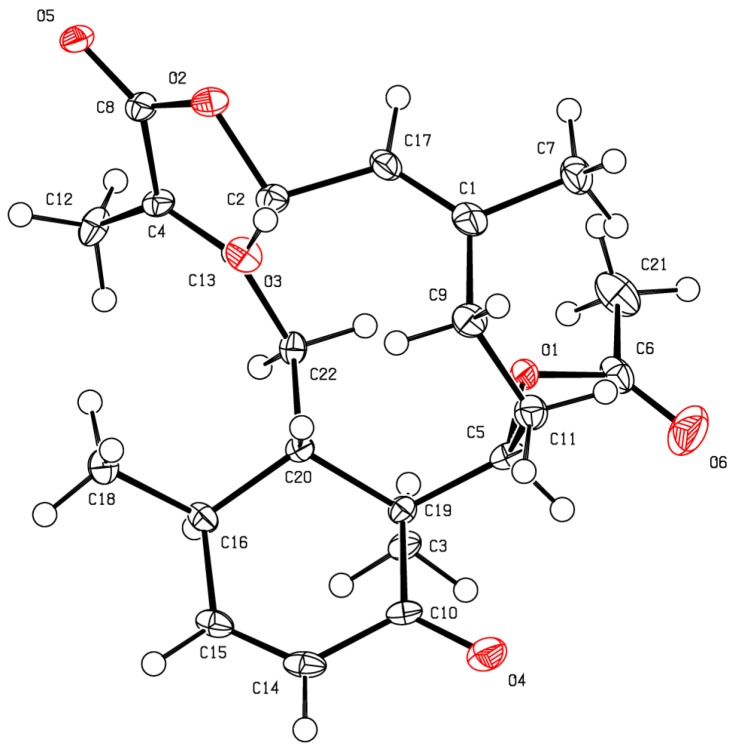
Asymmetric unit of bathyptilone B (**2**). Anisotropic displacement parameters were drawn at 50% probability.

As for bathyptilone A (**1**), bathyptilone B (**2**) was evaluated by 2D-NOE to determine the configuration of the asymmetric centers. H_3_-15 (δ_H_ 1.11) correlates to both H-2 (δ_H_ 5.89) and H-11 (δ_H_ 2.45), placing them on the same face of the bicyclic ring system, while H-10 (δ_H_ 3.43) only correlated to H-3a (δ_H_ 3.93). This evaluation supported the usual observation of the *trans* configured C-1/10 bridge in briarane-type scaffolds. X-ray crystallography (Figure 4) corroborated these assignments.

**Figure 5:**
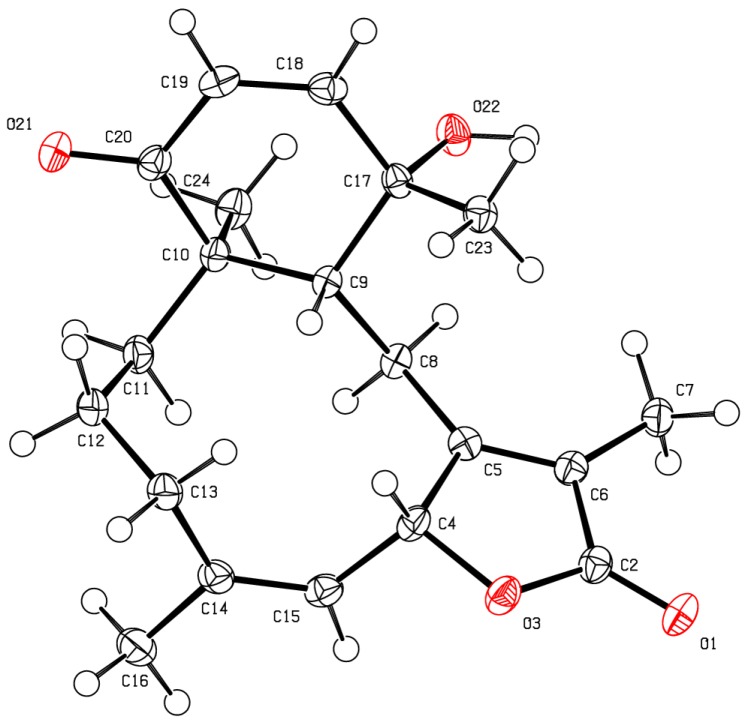
Asymmetric unit of bathyptilone C (**3**). Anisotropic displacement parameters were drawn at 50% probability.

Analysis of the crystalline bathyptilone C (**3**) with HRESIMS and 1D ^1^H and ^13^C NMR spectra yielded a molecular formula of C_20_H_26_O_4_. The ^1^H NMR spectrum of **3** showed shifts similar to compound **1** with the addition of a methine at δ_H_ 5.66 (H-7). The ^13^C NMR spectrum was closely matched to that of bathyptilone A, with the major difference being the upfield shift of C-7 (δ_C_ 79.2), which indicated the replacement of the bathyptilone A hemiacetal functionality by an alcohol function. Stereochemical analysis using NOE correlations, as well as comparison of shifts to bathyptilone A (**1**), established bathyptilone C as the C-7 reduced analog of **1**, an assignment which was confirmed by X-ray analysis (Figure 5).

**Figure 6:**
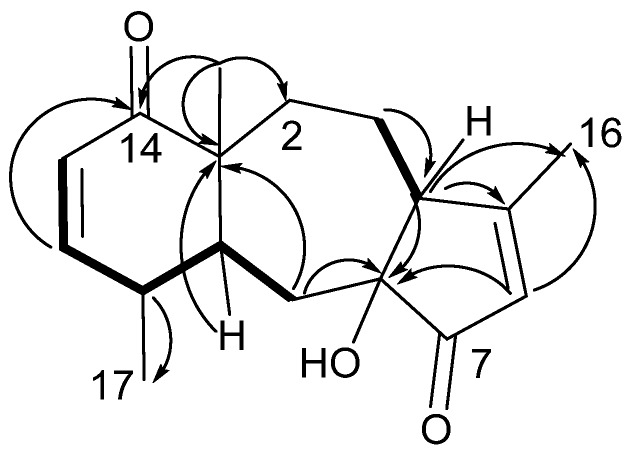
Key COSY (**▬**) and HMBC (→) correlations of enbepeanone A (**4**).

A fourth compound (**4**) isolated from *Anthoptilum grandiflorum* was determined to have the molecular formula C_17_H_22_O_3_ based on HRESIMS. 1D ^1^H and ^13^C NMR spectra (Table 3) suggested that compound **4** was a terpenoid, despite the odd carbon count. There were seven degrees of unsaturation that were accounted for by the presence of two ketone carbonyls, two olefins and a tricyclic ring system. Initially, COSY was used to identify two isolated spin systems. Starting with the most deshielded protons, δ_H_ 6.57 (H-12) and δ_H_ 6.07 (H-13) were coupled and H-12 further coupled to aliphatic methine δ_H_ 2.08 (H-11). The spin system bifurcated at H-11, with the system terminating in one direction with a methyl group at δ_H_ 1.09 (H_3_-17) but extending the system in the other direction to a methylene at δ_H_ 1.35 (H-10). The second spin system established by COSY was found as a methine at δ_H_ 2.20 (H-4) coupled to δ_H_ 1.20 (H-3a). Proton H-4 was extended by HMBC to an α,β-unsaturated ketone based on correlation to δ_C_ 177.5 (C-5), δ_C_ 133.2 (C-6), δ_C_ 205.4 (C-7), and δ_C_ 99.5 (C-8). Proton H-4 further correlated in the HMBC spectrum to a vinyl methyl at δ_H_ 2.09 (H_3_-16). The remaining methyl group, δ_C_ 19.3 (C-15), was assigned based on HMBC correlations to δ_C_ 52.8 (C-1), δ_C_ 31.9 (C-2) and δ_C_ 202.9 (C-14). The complete two-dimensional structure of **4** was completed by connecting the first COSY spin system to C-1, C-8 and C-14, as depicted in Figure 6. 

**Figure 7:**
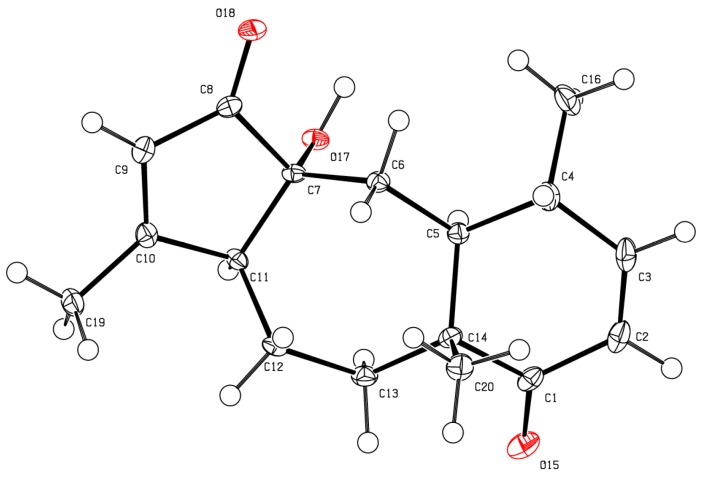
Asymmetric unit of enbepeanone A (**4**). Anisotropic displacement parameters were drawn at 50% probability.

The stereochemistry of enbepeanone A (**4)** was assigned from NOESY. Proton H-10 showed a strong correlation to the methyl doublet, which indicated their proximity; however, the methyl singlet of H-15 did not have a NOESY correlation to H-10 as noted for the briarane scaffolds in the bathyptilones. The cyclopentenone stereocenters, and their relationship to the cyclohexenone, were established by X-ray crystallographic analysis (Figure 7). The crystallographic metadata (Table S4) facilitated the assignment of the absolute configuration of enbepeanone A as shown.

**Scheme 2:**
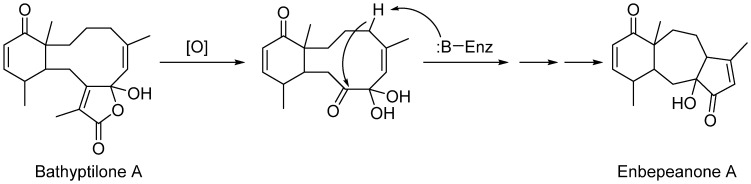
Hypothetical biochemical conversion of briarane bathyptilone A (**1**) into the enbepeanone skeleton.

Enbepeanone A (**4**) is a trinorditerpene seemingly derived from the oxidation of a briarane skeleton such as bathyptilone A (**1**). Oxidative cleavage of the C-8 pendant isopropyl group from **1** provides an intermediate suited to enzyme-catalyzed cyclopentanulation that would produce the enbepeanone A skeleton (Scheme 2). This is the first example of such a trinorditerpene bearing this carbon skeleton.

**Figure 8:**
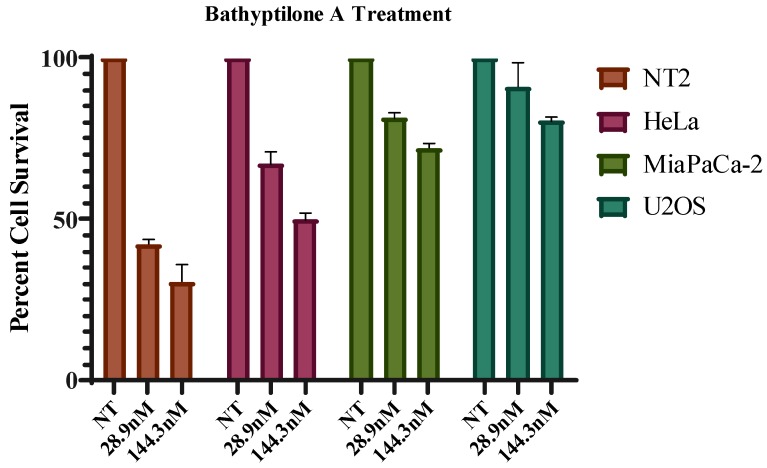
Bioactivity of bathyptilone A (**1**) against NT2, HeLa, MiaPaca-2 and U2OS cell lines. NT, no treatment.

Several mammalian cell types were analyzed for sensitivity to these diterpenes (Figure 8). Of the compounds tested, bathyptilone A (**1**) was highly toxic to a pluripotent embryonal carcinoma cell line, NTera-2 (NT2). This cell line was isolated from a lung metastasis of testicular cancer; however, these cells exhibit many properties of precursor neuronal cells like the formation of neurons and the presence of homeobox clusters in the genome [18]. Due to their similarity with developing neuronal cells, this cell line is accepted as a valid system for the assessment of neurotoxicity [19]. Compound **1** effectively killed the NT2 cells at with an IC_50_ of 29 nM. A recent publication describes the promising antiproliferative effect of gedunin with an IC_50_ as low as 13.5 µM against NT2 cells [20]. Compounds **2**–**4** were also tested against the NT2 cell line, but their activities were not significant. Based on this finding, we speculate that bathyptilone A may prove to be an effective therapeutic strategy for specifically targeting neuronal cancers. 

## 3. Experimental Section

### 3.1. General Experimental Procedure

Optical Rotations were measured by a Rudolph Instruments (Hackettstown, NJ, USA) AutoPol IV polarimeter at 589 nm. UV absorptions were measured by an Agilent (Santa Clara, CA, USA) Cary 60 UV-Vis spectrophotometer; wavelengths are recorded in nm. IR spectra were recorded with a PerkinElmer (Waltham, MA, USA) Spectrum Two with a UATR (single-reflection diamond) sample introduction system; peaks are reported in cm^−1^. NMR spectra were recorded at 298 K on an Agilent (Santa Clara, CA, USA) Varian Direct Drive 500 or Varian Direct Drive 800 MHz NMR spectrometers. Chemical shifts are reported with the use of the residual CDCl_3_ signals (δ_H_ 7.27 ppm; δ_C_ 77.0 ppm) as internal standards for ^1^H and ^13^C NMR spectra. The ^1^H and ^13^C NMR assignments were supported by COSY, HSQC, HMBC, and NOESY experiments. The high-resolution mass spectra were recorded on an Agilent (Santa Clara, CA, USA) 6230 TOF LC/MS. Semi-preparative and analytical HPLC was performed on a Shimadzu (Columbia, MD, USA) LC-20 AT system equipped with an evaporative light-scattering detector (ELSD) and a ultraviolet detector using a Luna column (5 μm, 250 × 10 mm) and a YMC (Kyoto, Japan) packed column (10 µm, 150 × 4 mm). MPLC was performed on a Teledyne Isco (Lincoln NE) CombiFlash Rf 200i equipped with an ELSD using a RediSep Rf silica 120 g flash column with silica gel 230–400 mesh ASTM used to load the sample. 

### 3.2. Animal Material

Specimens of the pennatulacean octocoral *Anthoptilum grandiflorum* were collected in April 2013, north of Burdwood Bank (54°14′56.9″ S, 59°00′00.6″ W), by trawling on the R/V Nathaniel B. Palmer from depths of between 662 and 944 meters. A specimen voucher is retained at the Scripps Institution of Oceanography Benthic Invertebrate collection (SIO-BIC Co2892, field number S22350). A piece of tissue from this voucher was extracted with a DNeasy kit (Qiagen) and was amplified for the mitochondrial msh1 (muts homolog) to confirm identification. We used the primers ND42599F/mut3458R [21,22] to produce amplicons and outsourced these for sequencing (AGRF Perth node). The resulting bi-directional sequence was compared to other available Pennatulacea sequences in GenBank using a Maximum-Likelihood analysis at the W-IQ-tree server [23]. W-IQ-Tree incorporates the ModelTest [24], which selected the K3P + G4 as the best-fit model, and which is automatically applied. The tree topology was assessed using 1000 ultrafast bootstrap replicates and shows that our specimen has an identical msh1 sequence to a specimen of *Anthoptilum grandiflorum* from Greenland (Supplementary information) [25]. The species has been recorded as having a worldwide distribution [26], although this study represents the southernmost record of this species.

### 3.3. Isolation of Bathyptilones and Enbepeanone

The *Anthoptilum grandiflorum* was immediately frozen after collection and later freeze-dried at our lab. The lyophilized organism (84.7 g) was then broken into smaller pieces and exhaustively extracted via 3 cycles of DCM (3.5 L) in a Soxhlet extractor. The crude extract (11.7 g) was partitioned through liquid–liquid extraction using DCM and water. The organic layer (10.4 g) was then concentrated, resulting in a brownish residue, and then re-suspended in DCM to be dried onto silica gel for flash chromatography using MPLC. A concentration gradient of hexane and ethyl acetate was extended over a period of time to elute 8 fractions (A–H). Fraction E (191.0 mg) eluted in 9:11 hexane/ethyl acetate and was subjected to further purification in normal phase HPLC on a silica gel column to afford 11 fractions. Fractions 4, 6, and 9 afforded the tricyclic diterpene compounds, bathyptilone A (**1**, 3.6 mg), B (**2**, 2.1 mg), C (**3**, 1.8 mg) and enbepeanone A (**4**, 0.7 mg) with further purification on a C_18_ reverse-phase HPLC column. The crystallization of these compounds occurred through slow evaporation using the mobile-phase solvents.

*Bathyptilone A* (**1**): Crystalline solid; [α]${}_{D}^{24}$ −18.3 (*c* 0.11, CHCl_3_); UV *λ*_max_ (EtOH) (log ε) 271 nm; IR 3650, 3356, 2980, 2646, 2322, 2286, 2166, 1738, 1656, 1446, 1378, 1212, 1114, 934, 892, 834, 754 cm^−1^; ^1^H NMR (500 MHz, CDCl_3_) and ^13^C NMR (125 MHz, CDCl_3_), see Table 1; HRESIMS *m/z* 347.1853 [M + H]^+^ (347.1859 calculated for C_20_H_27_O_5_).

*Bathyptilone B* (**2**): Crystalline solid; [α]${}_{D}^{24}$ −40.2 (*c* 0.11, CHCl_3_); IR 3346, 2980, 2886, 1736, 1662, 1456, 1378, 1230, 938, 752 cm^−1^; ^1^H NMR (800 MHz, CDCl_3_), see Table 2 and ^13^C NMR (200 MHz, CDCl_3_), see Table 2; HRESIMS *m/z* 411.1775 [M + Na]^+^ (411.1784 calculated for C_22_H_28_O_6_Na).

*Bathyptilone C* (**3**): Crystalline solid; [α]${}_{D}^{24}$ −16.2 (*c* 0.11, CHCl_3_); ^1^H NMR (800 MHz, CDCl_3_), see Table 2 and ^13^C NMR (200 MHz, CDCl_3_), see Table 2; HRESIMS *m/z* 330.1842 [M + H]^+^ (330.1831 calculated for C_20_H_27_O_4_).

*Enbepeanone A* (**4**): Crystalline solid; [α]${}_{D}^{24}$ −11.0 (*c* 0.11, CHCl_3_); IR 3424, 3190, 2646, 2322, 1650, 1020, 742, 512, 456, 414 cm^−1^; ^1^H NMR (500 MHz, CDCl_3_) and ^13^C NMR (125 MHz, CDCl_3_), see Table 3; HRESIMS *m/z* 275.1640 [M + H]^+^ (275.1647 calculated for C_17_H_23_O_3_).

### 3.4. X-Ray Crystallography

The X-ray diffraction data were measured on a Bruker D8 Venture PHOTON 100 CMOS system (Madison, WI, USA) equipped with a Cu K_α_ INCOATEC ImuS micro-focus source (*λ* = 1.54178 Å). Indexing was performed using Apex3 [27]. Data integration and reduction were performed using SaintPlus 6.01 [28]. Absorption correction was performed by a multi-scan method implemented in SADABS [29]. Space groups were determined using XPREP implemented in APEX3 [27]. Structures were solved using SHELXT [30] and refined using SHELXL-2017 [31,32,33] (full-matrix least-squares on F^2^) through the OLEX2 interface program [34]. All non-hydrogen atoms were refined anisotropically. The hydrogen atoms of –OH groups were freely refined. The remaining hydrogen atoms were placed in geometrically calculated positions and were included in the refinement process using a riding model with isotropic thermal parameters. Crystal data and refinement conditions are shown in Supplementary Tables S1–S5. The absolute configuration for compounds **1** and **3** was established based on the Flack parameter value and verified additionally with Bijvoet–Pair Analysis and Bayesian Statistics Methods [35,36] (Table S5). P2 ≈ 1 for all cases and this represents the probability that the current model is correct assuming the possibility that one out of two enantiomers is present. The results of the absolute configuration assignments for compound **2** are not conclusive**.**

Crystallographic data for the structures reported in this paper have been deposited with the Cambridge Crystallographic Data Centre as supplementary publication nos. CCDC 1,944,295 for **1**, CCDC 1,944,292 for **2**, CCDC 1,944,293 for **3** and CCDC 1,944,294 for **4**. Copies of the data can be obtained, free of charge, on application to the Director, CCDC, 12 Union Road, Cambridge CB2 1EZ, UK.

*Crystallographic data for bathyptilone A (**1**).* C_20_H_26_O_5_, M = 346.41, crystal size 0.093 × 0.038 × 0.034 mm^3^, orthorhombic, *a =* 8.7926(2) Å, *b* = 12.2611(2) Å, *c* = 16.4222(3) Å, α = β = γ = 90°, V = 1770.43 (6) Å^3^, T = 100.01 K, space group P2_1_2_1_2_1_, Z = 4, D_calcd_ = 1.300 g/cm^3^, µ = 0.753 mm^−1^, F (000) = 744.0, 3123 independent reflections (R_int_ = 0.0453, R_sigma_ = 0.0213). The final R_1_ = 0.0288 (I ≥ 2σ (I)), *w*R_2_ = 0.0674 (I ≥ 2σ (I)), R_1_ = 0.0312 (all data), *w*R_2_ = 0.0688 (all data), S = 1.088. The Flack parameter was −0.04 (6).

*Crystallographic data for bathyptilone B (**2)**.* C_22_H_28_O_6_, M = 388.44, crystal size 0.04 × 0.02 × 0.005 mm^3^, monoclinic, *a =* 9.5139(4) Å, *b* = 10.3255(5) Å, *c* = 10.4615(4) Å, α = γ = 90°, β = 90.924° (3), V = 1027.56 (8) Å^3^, T = 100.02 K, space group P2_1_, Z = 2, D_calcd_ = 1.255 g/cm^3^, µ = 0.744 mm^−1^, F (000) = 416.0, 3614 independent reflections (R_int_ = 0.1118, R_sigma_ = 0.1052). The final R_1_ = 0.0576 (I≥2σ (I)), *w*R_2_ = 0.1076 (I ≥ 2σ (I)), R_1_ = 0.0859 (all data), *w*R_2_ = 0.1184 (all data), S = 1.041. The Flack parameter was 0.2 (3).

*Crystallographic data for bathyptilone C (**3**).* C_20_H_26_O_4_, M = 330.41, crystal size 0.467 × 0.058 × 0.055 mm^3^, orthorhombic, *a* = 8.2529(2) Å, *b* = 10.8806(3) Å, *c* = 19.0878(5) Å, α = β = γ = 90°, V = 1714.02 (8) Å^3^, T = 99.99 K, space group P2_1_2_1_2_1_, Z = 4, D_calcd_ = 1.280 g/cm^3^, µ = 0.707 mm^−1^, F (000) = 712.0, 3597 independent reflections (R_int_ = 0.0725, R_sigma_ = 0.0359). The final R_1_ = 0.0356 (I ≥ 2σ (I)), *w*R_2_ = 0.0852 (I ≥ 2σ (I)), R_1_ = 0.0398 (all data), *w*R_2_ = 0.0885 (all data), S = 1.071. The Flack parameter was 0.03 (9).

*Crystallographic data for enbepeanone A (**4**).* C_17_H_22_O_3_, M = 274.34, crystal size 0.2 × 0.06 × 0.02 mm^3^, orthorhombic, *a =* 7.1483(2) Å, *b* = 7.7391(2) Å, *c* = 25.4330(7) Å, α = β = γ = 90°, V = 1406.99 (7) Å^3^, T = 100.0 K, space group P2_1_2_1_2_1_, Z = 4, D_calcd_ = 1.295 g/cm^3^, µ = 0.698 mm^−1^, F (000) = 592.0, 2884 independent reflections (R_int_ = 0.0551, R_sigma_ = 0.0432). The final R_1_ = 0.0374 (I ≥ 2σ (I)), *w*R_2_ = 0.0789 (I ≥ 2σ (I)), R_1_ = 0.0455 (all data), *w*R_2_ = 0.0825 (all data), S = 1.105. The Flack parameter was −0.03 (13).

### 3.5. Biological Assays

*Ntera-2, MiaPaca-2, HeLa and U2OS cell lines.* Several cell lines were tested for sensitivity to the tricyclic diterpenes, the cell lines tested were HeLa (ATCC CCL-2™), MiaPaCa-2 (ATCC CRL-1420™, U2OS (ATCC HTB-96™), and NT2 (ATCC CRL-1973™). All cells used were seeded into 96-well plates (~25 cells per well) and allowed to attach for 48 h under normal growth conditions (described below). Compounds were administered at the indicated concentrations and the cells were allowed to grow for 10 days. All cell lines used were grown in Dulbecco’s Modification of Eagle’s Medium (DMEM), with 4.5 g/L glucose and l-glutamine (Corning, 10-107-CV) supplemented with 10% Fetal Bovine Serum (Seradigm, 3100-500) and 1% penicillin–streptomycin–glutamine (HyClone, SV30082.01). All cell lines were grown at 37 °C with 5% CO_2_ in a FORMA Series II Water Jacket CO_2_ Incubator (Thermo). After the growth period, the cells were fixed with a 10% methanol + 10% acetic acid solution for 15 minutes at room temperature. The fixed cells were stained with crystal violet for 5 minutes and distained by washing with water. The cells were dried overnight. Crystal violet was removed from the cells with Sorensen buffer (0.1M sodium citrate, 50% ethanol), the colorimetric intensity of each solution was quantified using Gen5 software on a Synergy 2 (BioTek, Winooksi, VT, USA) plate reader (OD at 595 nm). Error bars are representative of 3 independent experiments.

## Figures and Tables

**Table 1:** NMR Spectroscopic Data for Bathyptilone A (**1**).

Position	^13^C, type	^1^H (*J* in Hz)	HMBC
1	50.2, C		
2	21.8, CH_2_	a 1.54, m b 1.84, m	14 1, 4
3	31.6, CH_2_	a 1.52, m b 1.98, m	2 1, 2, 4, 10, 15
4	29.5, CH_2_	a 1.85, m b 3.52, br t	5, 6
5	145.3, C		
6	124.1, CH	5.35, s	4, 5, 7, 16
7	106.2, C		
8	160.7, C		
9	22.7, CH_2_	a 2.47, d (15.8) b 3.25, dd (9.5, 15.3)	1, 8, 10, 11, 17 7, 8, 10, 11, 17
10	41.4, CH	3.14, d (9.5)	1, 8, 9, 11, 14, 15
11	69.3, C		
12	151.7, CH	6.63, d (10.0)	10, 11, 14, 20
13	127.4, CH	5.97, d (10.0)	1, 11
14	203.7, C		
15	24.2, CH_3_	1.16, s	1, 2, 3, 10, 14
16	23.5, CH_3_	1.78, s	4, 5, 6
17	125.8, C		
18	171.7, C		
19	9.1, CH_3_	1.95, s	8, 17, 18
20	30.4, CH_3_	1.35, s	10, 11, 12
	7, 11-OH	3.52, br	

**Table 2:** ^1^H and ^13^C NMR Data for Bathyptilone B (**2**) and Bathyptilone C (**3**).

Position	Bathyptilone B (2)		Bathyptilone C (3)	
	C, type ^a,c^	^1^H (*J* in Hz) ^b^	C, type	^1^H (*J* in Hz)
1	52.5, C		50.5, C	
2	77.9, CH	5.89, dd (4.8, 12.9)	31.5, CH_2_	a 1.90, m b 1.56, m
3	27.6, CH_2_	a 3.93, dt (3.2, 14.7) b 1.89, m	21.8, CH_2_	a 1.95, m b 1.52, m
4	26.3, CH_2_	a 1.99, m b 2.32, m	30.3, CH_2_	a 2.60, td (13.5, 5.3) b 2.03, m
5	146.0, C		141.2, C	
6	122.8, CH	5.26, s	122.4, CH	5.00, br d (8.6)
7	106.3, C		79.2, CH	5.66, br d (8.2)
8	161.3, C		159.4, C	
9	28.4, CH_2_	a 3.64, m b 2.40, m	22.4, CH_2_	a 3.28, br dd (15.6, 9.7) b 2.34, br d (15.6)
10	41.4, CH	3.34, t (8.5)	44.8, CH	2.42, br d (9.3)
11	38.0, CH	2.45, m	68.9, C	
12	154.8, CH	6.64, br d (10.3)	151.2, CH	6.62, br d (9.7)
13	127.0, CH	5.94, d (10.3)	127.6, CH	6.00, br d (9.7)
14	201.4, C		203.2, C	
15	16.7, CH_3_	1.11, s	24.0, CH_3_	1.14, s
16	23.2, CH_3_	1.77, s	22.7, CH_3_	1.74, s
17	124.3, C		122.9, C	
18	171.7, C	1.91, s	163.9, C	
19	9.1, CH_3_		9.1, CH_3_	1.94, s
20	18.8, CH_3_	1.14, d (6.5)	30.8, CH_3_	1.27, s
21	169.5, C			
22	21.2, CH_3_	2.03, s		

**Table 3:** NMR data for enbepeanone A (**4**).

Position	^13^C, type	^1^H (*J* in Hz)
1	52.8, C	
2	31.9, CH_2_	1.14, m 1.39, m
3	19.5, CH_2_	1.20, m 1.30, m
4	46.3, CH	2.20, m
5	177.5, C	
6	133.2, CH	5.85, s
7	205.4, C	
8	99.5, C	
9	28.3, CH_2_	1.36, m 1.46, dd (12.4, 7.0)
10	32.2, CH	1.35, m
11	36.1, CH	2.08, m
12	153.6, CH	6.57, dd (10.9, 6.2)
13	128.0, CH	6.07, d (10.9)
14	202.9, C	
15	19.3, CH_3_	1.24, s
16	25.7, CH_3_	2.09, s
17	19.6, CH_3_	1.09, d (6.8)

## Supplementary Materials

The following are available online at https://www.mdpi.com/1660-3397/17/9/513/s1.
