# Supplementary material for: Bathyptilones: Terpenoids from an Antarctic Sea Pen, Anthoptilum grandiflorum (Verrill, 1879)
Source: Mar Drugs. 2019 Sep 1;17(9):513. doi: 10.3390/md17090513 (PMC6780369; doi:10.3390/md17090513)
Supplement: Supplementary file 1 [file marinedrugs-17-00513-s001.pdf]

Supporting Information

Bathyptilones: Terpenoids from an Antarctic sea pen, *Anthoptilum grandiflorum* (Verrill, 1879).

*Santana A.L. Thomas,[†] Anthony Sanchez,[‡] Younghoon Kee,[‡] Nerida G. Wilson,[§] Bill J. Baker[†,]**

Email: bjbaker@usf.edu; Phone: (813) 974-0274; Fax: (813) 905-9933

[†]Department of Chemistry, University of South Florida, Tampa, FL 33620, United States

[‡]Department of Cellular, Microbiology and Molecular Biology, University of South Florida, Tampa, FL 33620, United States

[§]Western Australia Museum, Perth, Western Australia, Australia

Figure S1. ^{1}H NMR spectrum for bathyptilone A (**1**) (500 MHz, $CDCl_3$) 3
Figure S2. ^{13}C NMR spectrum for bathyptilone A (**1**) (125 MHz, $CDCl_3$) 3
Figure S3. ^{1}H-^{1}H COSY NMR spectrum for bathyptilone A (**1**) (500 MHz, $CDCl_3$) 4
Figure S5. ^{1}H-^{13}C HSQC NMR spectrum for bathyptilone A (**1**) (800 MHz, $CDCl_3$) 4
Figure S4. ^{1}H-^{13}C HMBC NMR spectrum for bathyptilone A (**1**) (500 MHz, $CDCl_3$) 5
Figure S6. ^{1}H-^{1}H NOESY NMR spectrum for bathyptilone A (**1**) (500 MHz, $CDCl_3$) 5
Figure S7. HRESIMS spectrum for bathyptilone A (**1**) 6
Figure S8. ^{1}H NMR spectrum for bathyptilone B (**2**) (400 MHz, $CDCl_3$) 6
Figure S9. ^{13}C NMR spectrum for bathyptilone B (**2**) (125 MHz, $CDCl_3$) 6
Figure S10. ^{1}H-^{1}H COSY NMR spectrum for bathyptilone B (**2**) (500 MHz, $CDCl_3$) 7
Figure S11. ^{1}H-^{13}C HSQC NMR spectrum for bathyptilone B (**2**) (500 MHz, $CDCl_3$) 7
Figure S12. ^{1}H-^{13}C HMBC NMR spectrum for bathyptilone B (**2**) (500 MHz, $CDCl_3$) 8
Figure S13. ^{1}H-^{1}H ROESY NMR spectrum for bathyptilone B (**2**) (500 MHz, $CDCl_3$) 8
Figure S14. HRESIMS spectrum for bathyptilone B (**2**) 9
Figure S15. ^{1}H NMR spectrum for bathyptilone C (**3**) (800 MHz, $CDCl_3$) 9
Figure S16. ^{13}C NMR spectrum for bathyptilone C (**3**) (200 MHz, $CDCl_3$) 10
Figure S17. Zoomed-in region of Figure S16 from 0-75 ppm 10
Figure S18. Zoomed-in region of Figure S16 from 78-205 ppm 11

Figure S19. ^{1}H-^{1}H COSY NMR spectrum for bathyptilone C (**3**) (500 MHz, $CDCl_3$) 11
Figure S20. ^{1}H-^{13}C HSQC NMR spectrum for bathyptilone C (**3**) (800 MHz, $CDCl_3$) 12
Figure S21. ^{1}H-^{13}C HMBC NMR spectrum for bathyptilone C (**3**) (500 MHz, $CDCl_3$) 12
Figure S22. ^{1}H-^{1}H NOESY NMR spectrum for bathyptilone C (**3**) (800 MHz, $CDCl_3$)............................13
Figure S23. HRESIMS spectrum for bathyptilone C (**3**) 13
Figure S24. ^{1}H NMR spectrum for enbepeanone A (**4**) (500 MHz, $CDCl_3$)............................14
Figure S25. Zoomed-in region of Figure S24 from 0.9-1.53 ppm............................14
Figure S26. Zoomed-in region of Figure S24 from 1.59-6.7 ppm............................15
Figure S27. ^{13}C NMR spectrum for enbepeanone A (**4**) (125 MHz, $CDCl_3$) 15
Figure S28. Zoomed in region of Figure S27 from 0-50 ppm. 16
Figure S29. Zoomed in region of Figure S27 from 78-200 ppm 16
Figure S30. ^{1}H-^{1}H COSY NMR spectrum for enbepeanone A (**4**) (500 MHz, $CDCl_3$) 17
Figure S31. ^{1}H-^{13}C HSQC NMR spectrum for enbepeanone A (**4**) (500 MHz, $CDCl_3$) 17
Figure S32. ^{1}H-^{13}C HMBC NMR spectrum for enbepeanone A (**4**) (500 MHz, $CDCl_3$) 18
Figure S33. ^{1}H-^{1}H NOESY NMR spectrum for enbepeanone A (**4**) (500 MHz, $CDCl_3$)............................18
Figure S34. HRESIMS spectrum for enbepeanone A (**4**) 19
Figure S35. Maximum Likelihood tree topology comparing our *Anthoptilum* msh1 sequences with those available on Genbank............................20
Table S1. Crystal data and structure refinement for bathyptilone A **(1)**............................21
Table S2. Crystal data and structure refinement for bathyptilone B **(2)**............................21
Table S3. Crystal data and structure refinement for bathyptilone C **(3)**............................22
Table S4. Crystal data and structure refinement for enbepeanone A **(4)**............................22
Table S5. Bijvoet-Pair analysis and Bayesian statistics for (**1**) and (**4**)............................23

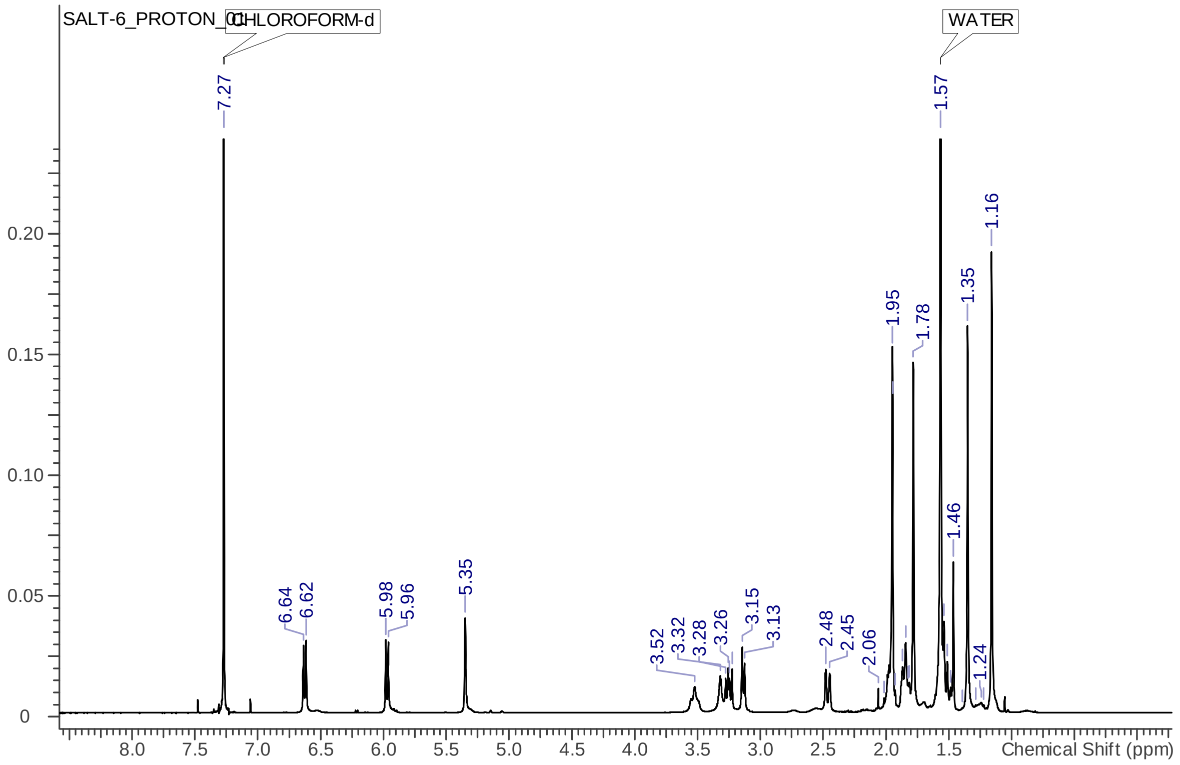

Figure S1. ^{1}H NMR spectrum for bathyptilone A (**1**) (500 MHz, $CDCl_3$).

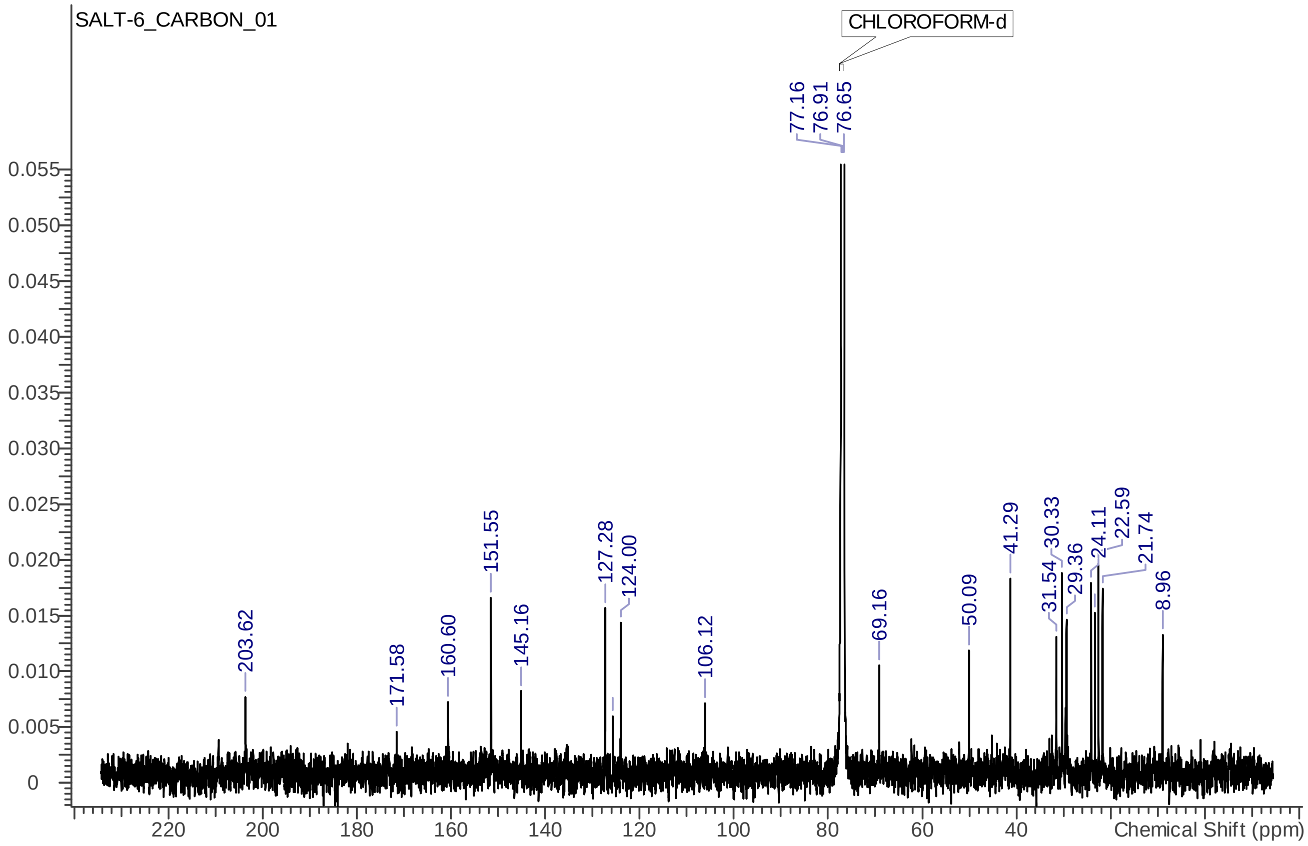

Figure S2. ^{13}C NMR spectrum for bathyptilone A (**1**) (125 MHz, $CDCl_3$).

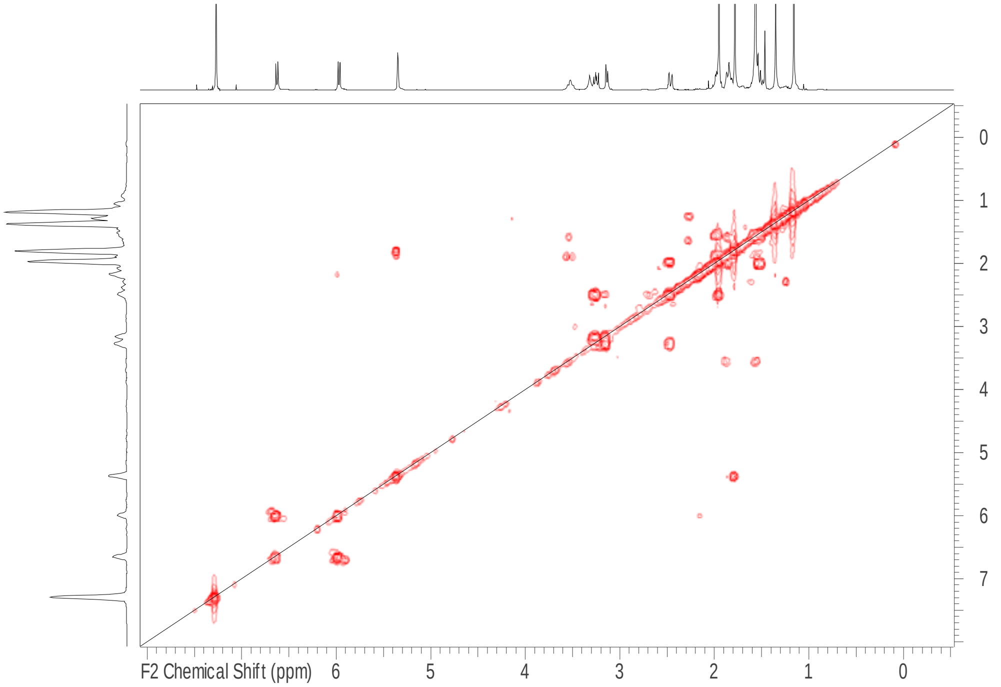

Figure S3. ^{1}H-^{1}H COSY NMR spectrum for bathyptilone A (**1**) (500 MHz, $CDCl_3$).

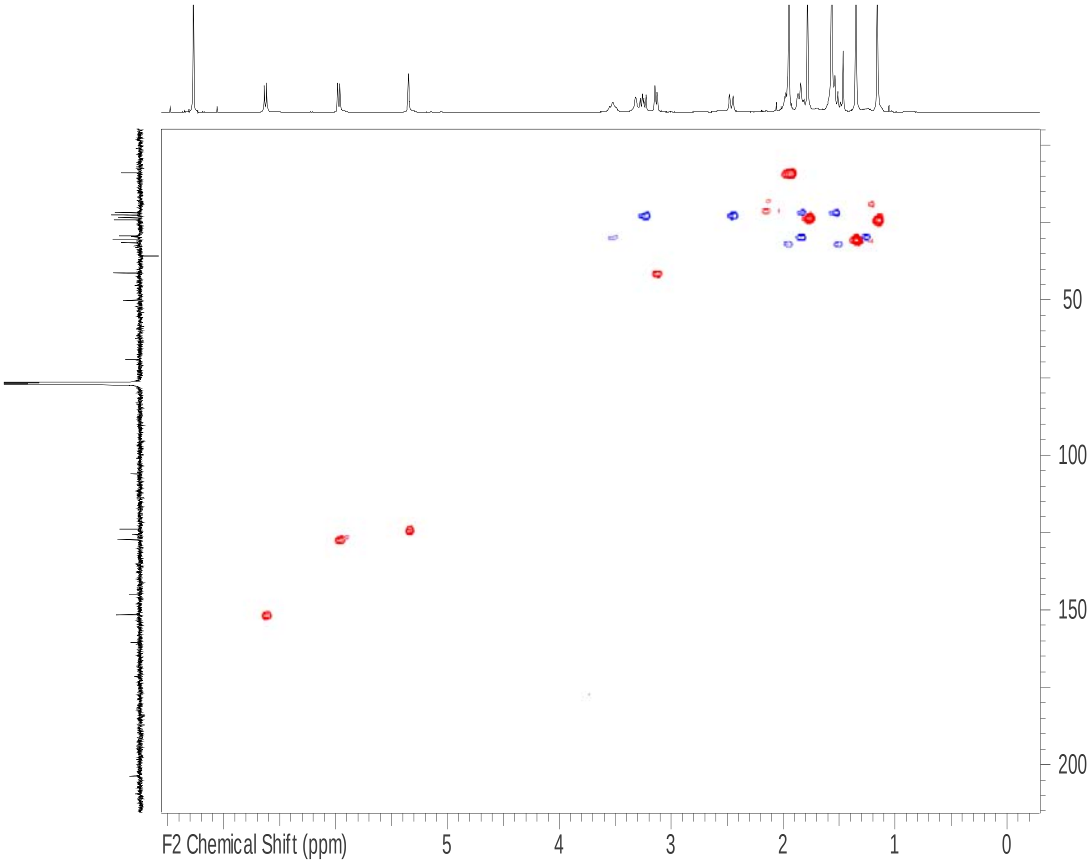

Figure S4. ^{1}H-^{13}C HSQC NMR spectrum for bathyptilone A (**1**) (500 MHz, $CDCl_3$).

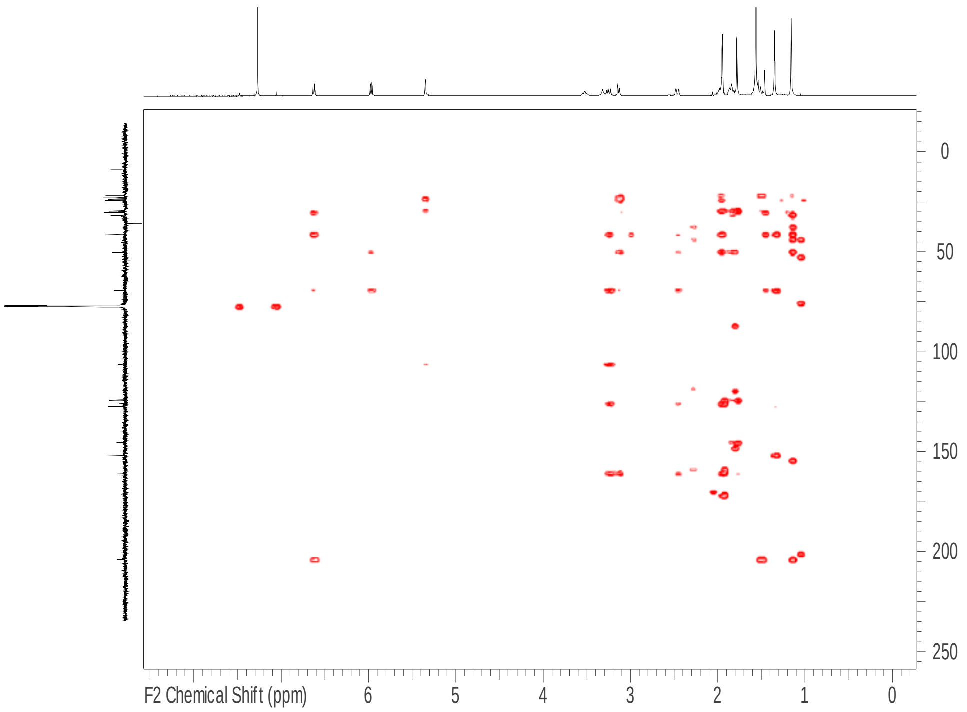

Figure S5. ^{1}H-^{13}C HMBC NMR spectrum for bathyptilone A (**1**) (500 MHz, $CDCl_3$).

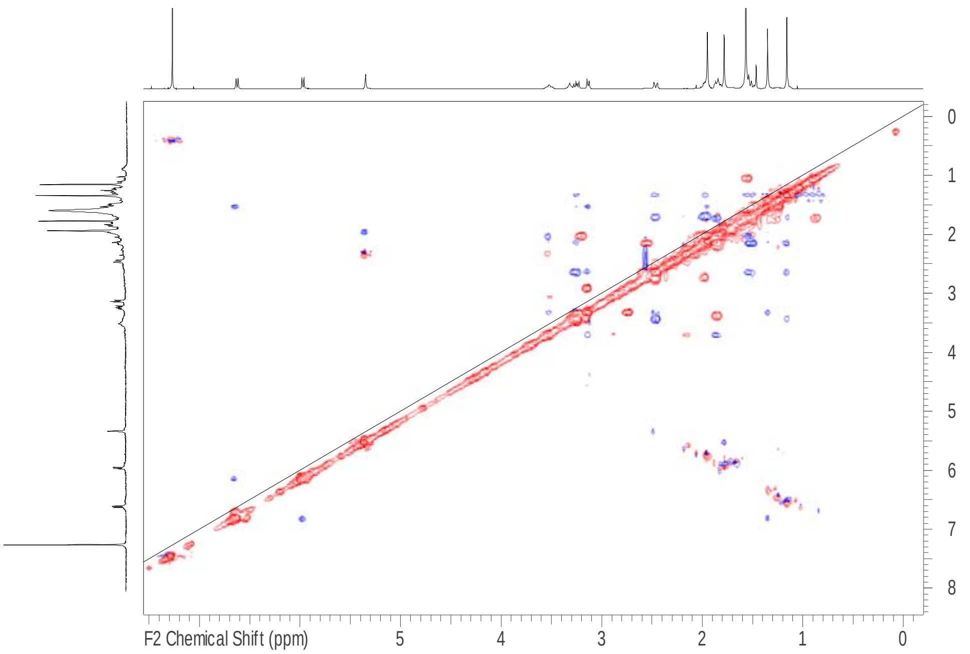

Figure S6. ^{1}H-^{1}H ROESY NMR spectrum for bathyptilone A (**1**) (500 MHz, $CDCl_3$).

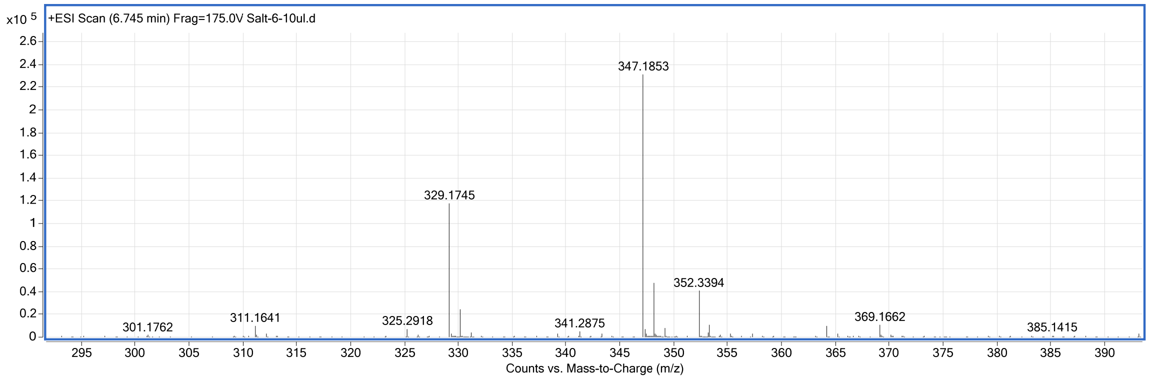

Figure S7. High resolution ESI-MS spectrum for bathyptilone A (**1**).

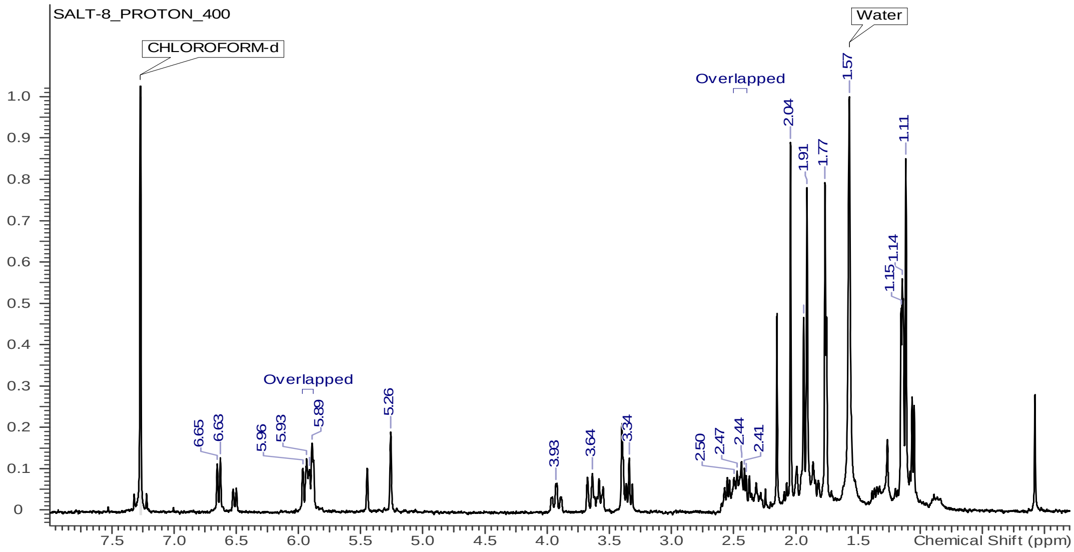

Figure S8. ^{1}H NMR spectrum for bathyptilone B (**2**) (400 MHz, $CDCl_3$). Minor peaks from inseparable derivative not marked.

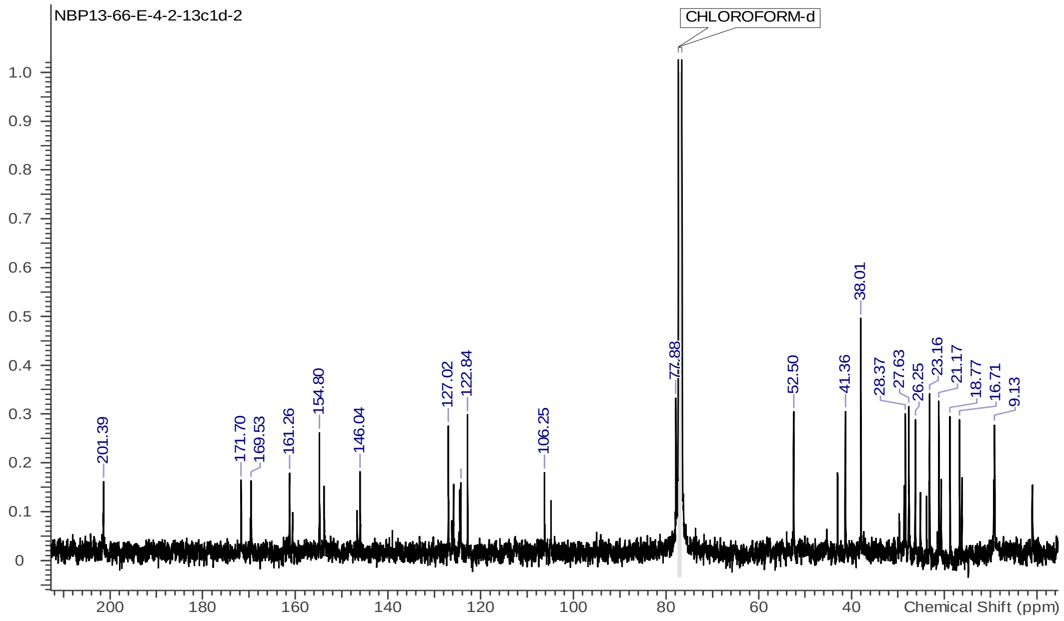

Figure S9. ^{13}C NMR spectrum for bathyptilone B (**2**) (125 MHz, $CDCl_3$). Minor peaks from inseparable derivative not marked.

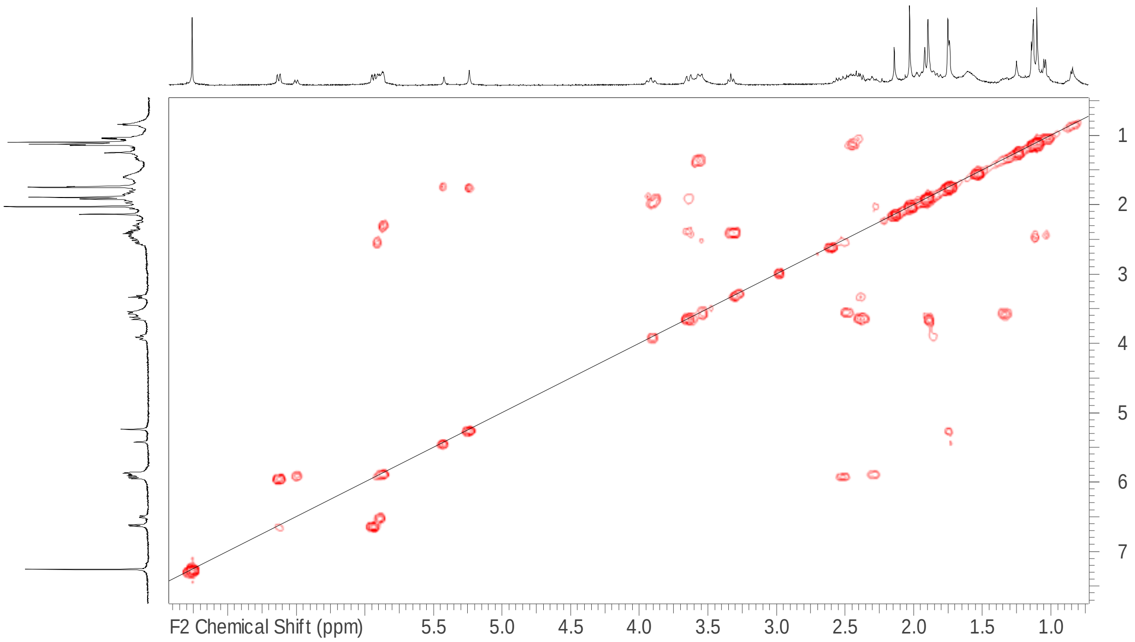

Figure S10. ^{1}H-^{1}H COSY NMR spectrum for bathyptilone B (**2**) (500 MHz, $CDCl_3$).

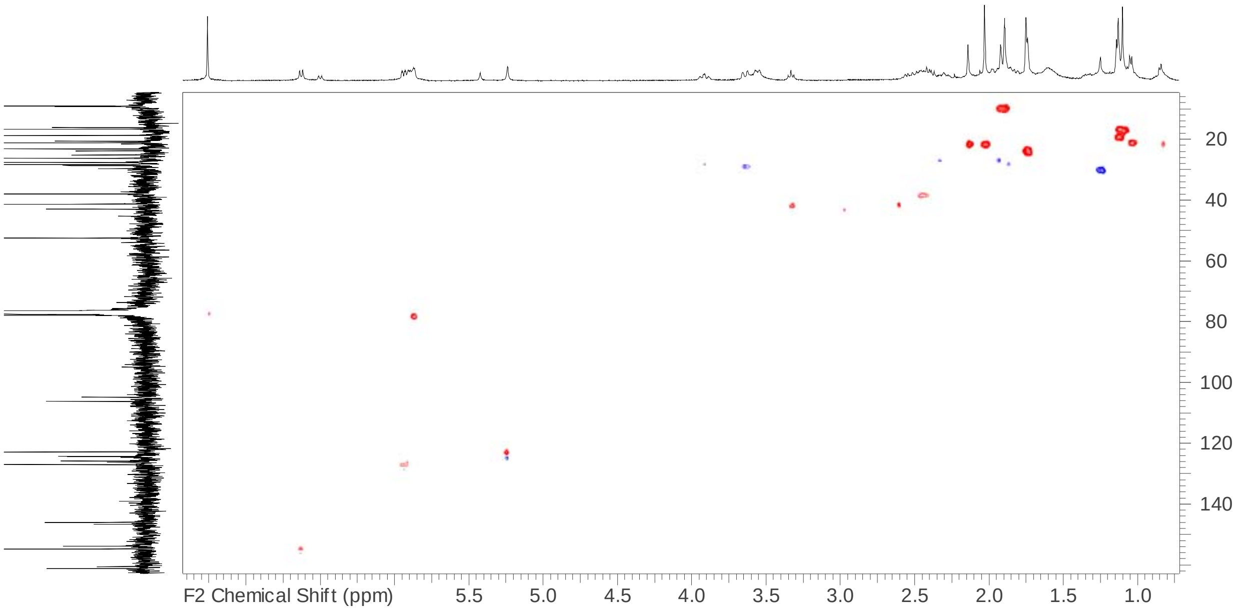

Figure S11. ^{1}H-^{13}C HSQC NMR spectrum for bathyptilone B (**2**) (500 MHz, $CDCl_3$).

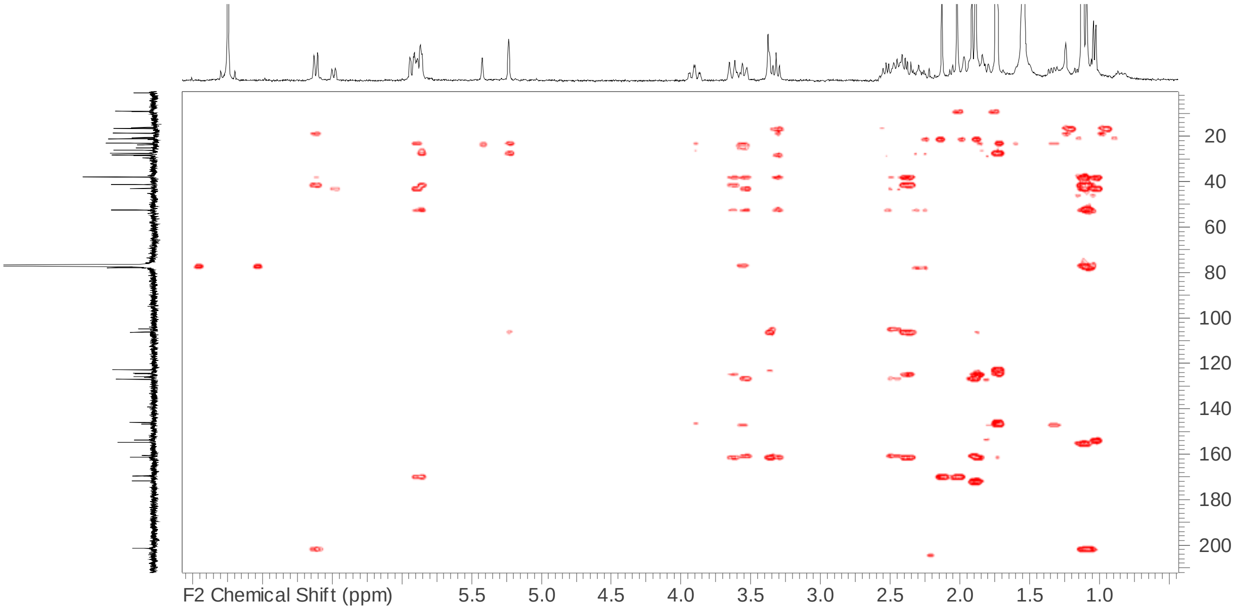

Figure S12. ^{1}H-^{13}C HMBC NMR spectrum for bathyptilone B (**2**) (500 MHz, $CDCl_3$).

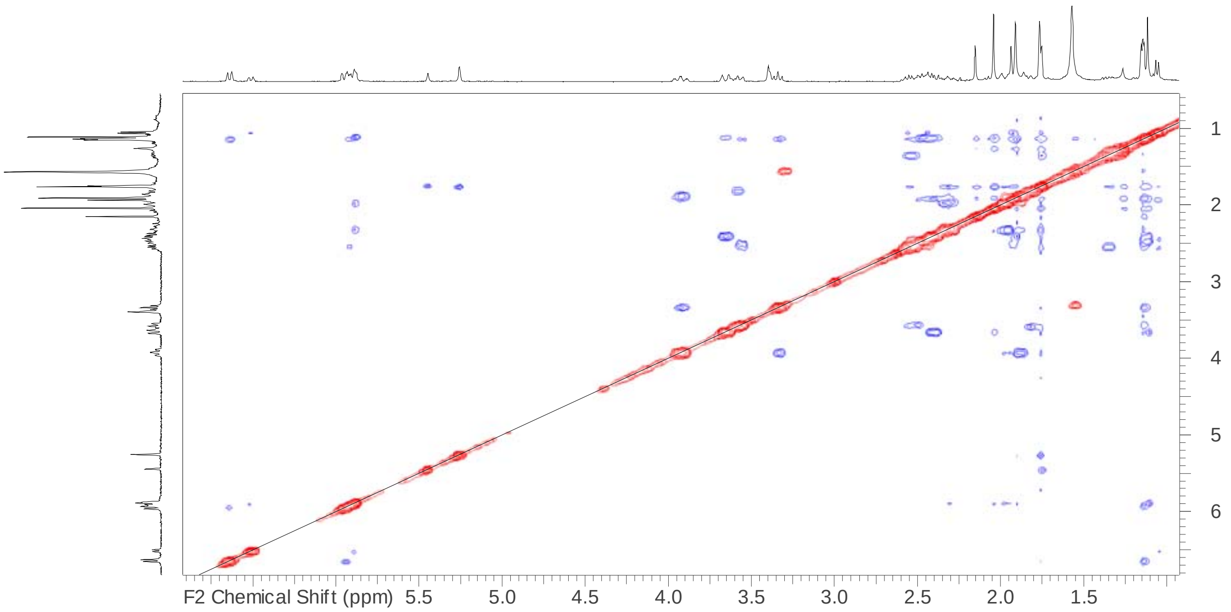

Figure S13. ^{1}H-^{1}H ROESY NMR spectrum for bathyptilone B (**2**) (500 MHz, $CDCl_3$).

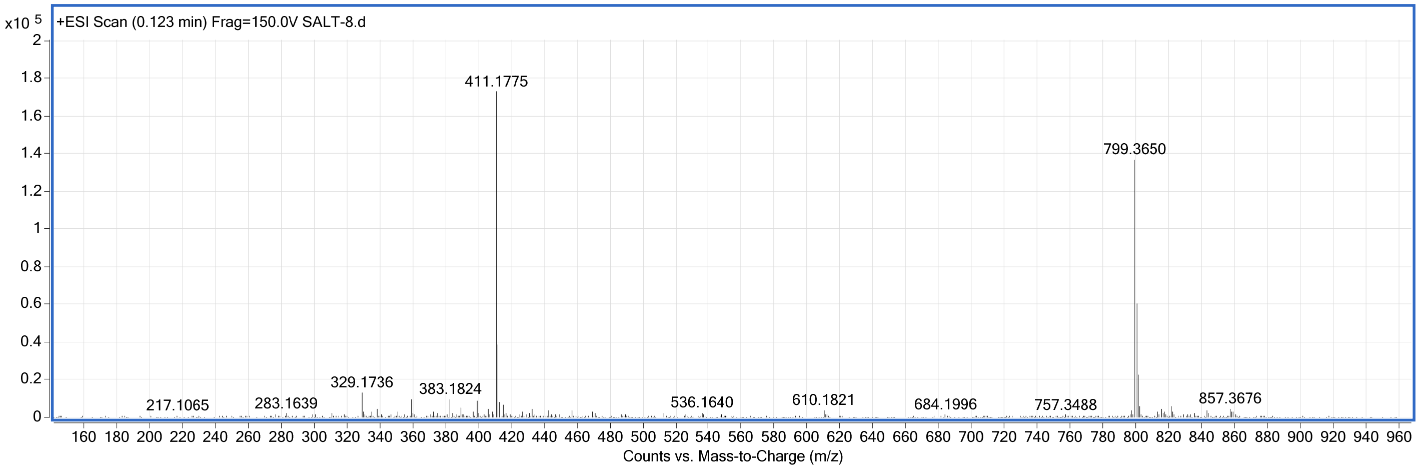

Figure S14. High resolution ESI-MS spectrum for bathyptilone B (**2**).

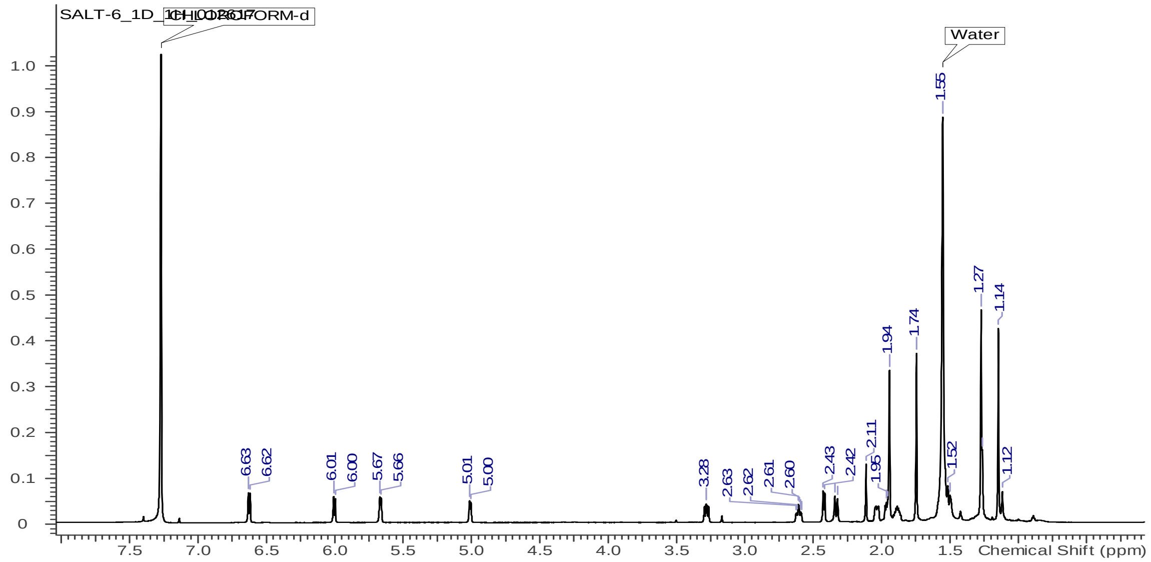

Figure S15. ^{1}H NMR spectrum for bathyptilone C (**3**) (800 MHz, $CDCl_3$).

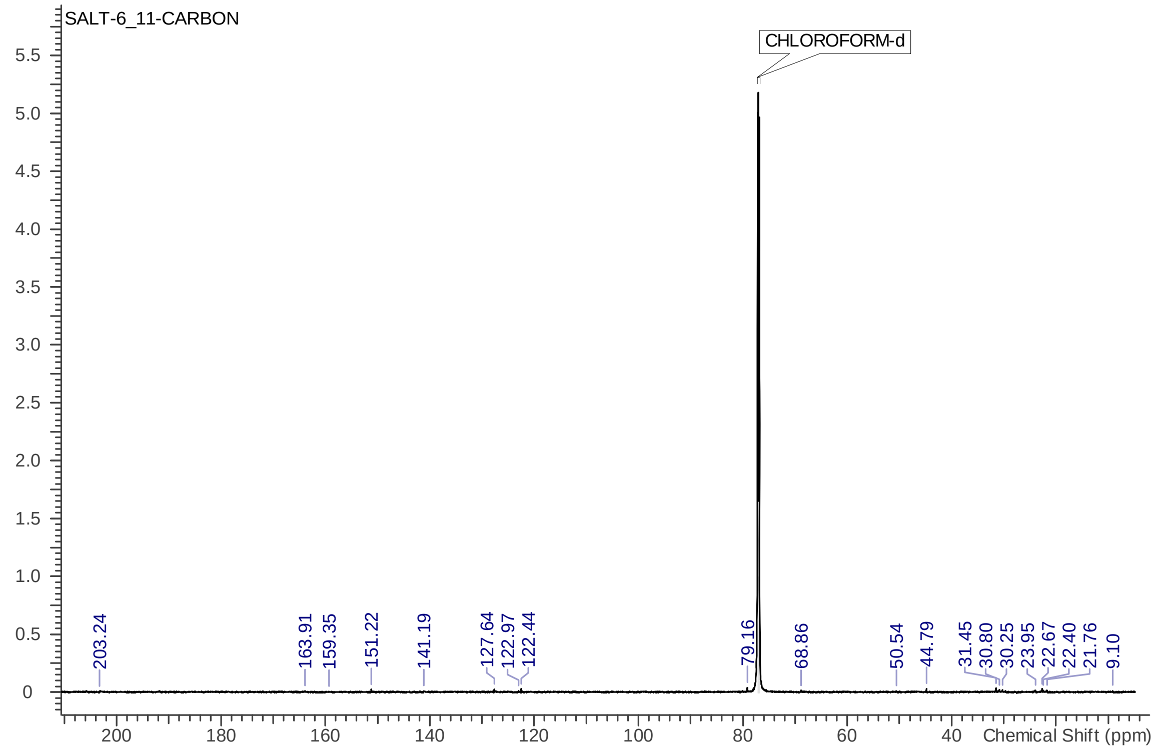

Figure S16. ^{13}C NMR spectrum for bathyptilone C (**3**) (200 MHz, $CDCl_3$).

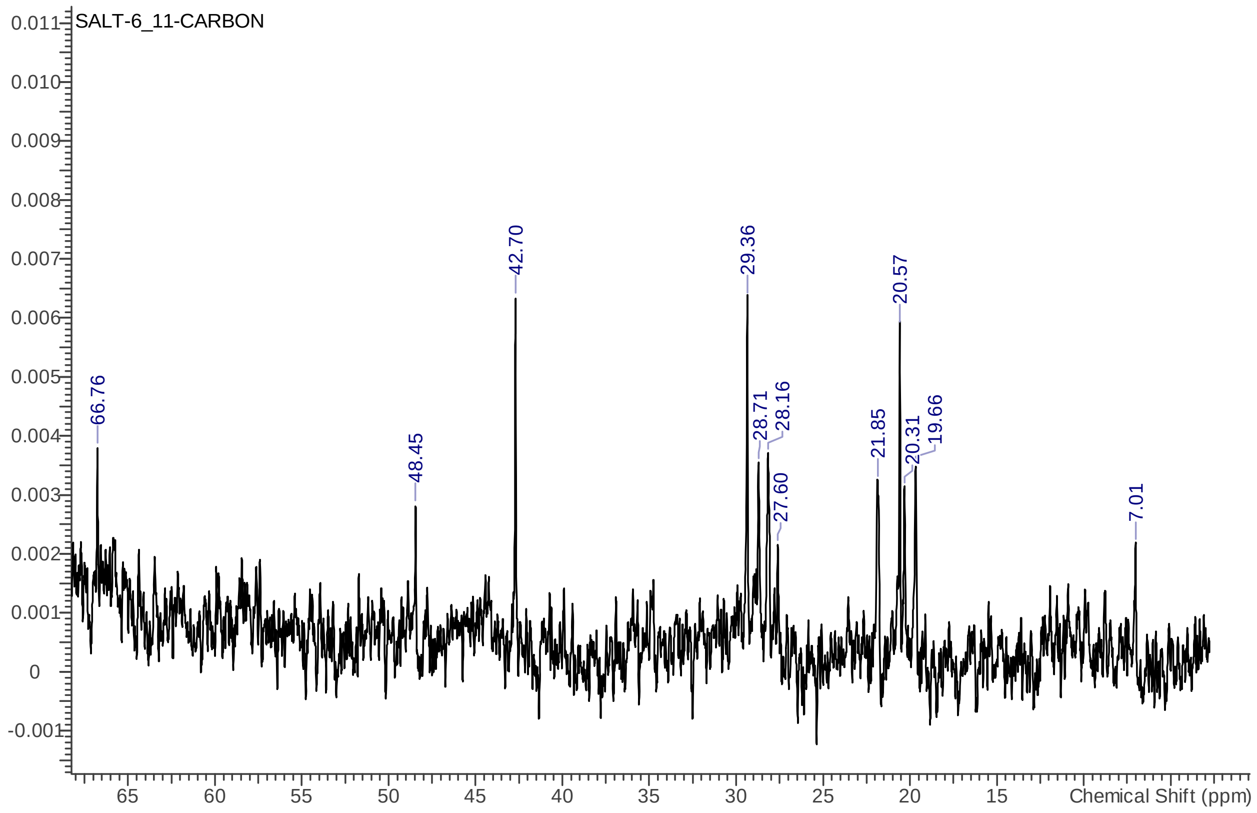

Figure S17. Zoomed-in region of Figure S16 from 0-75 ppm.

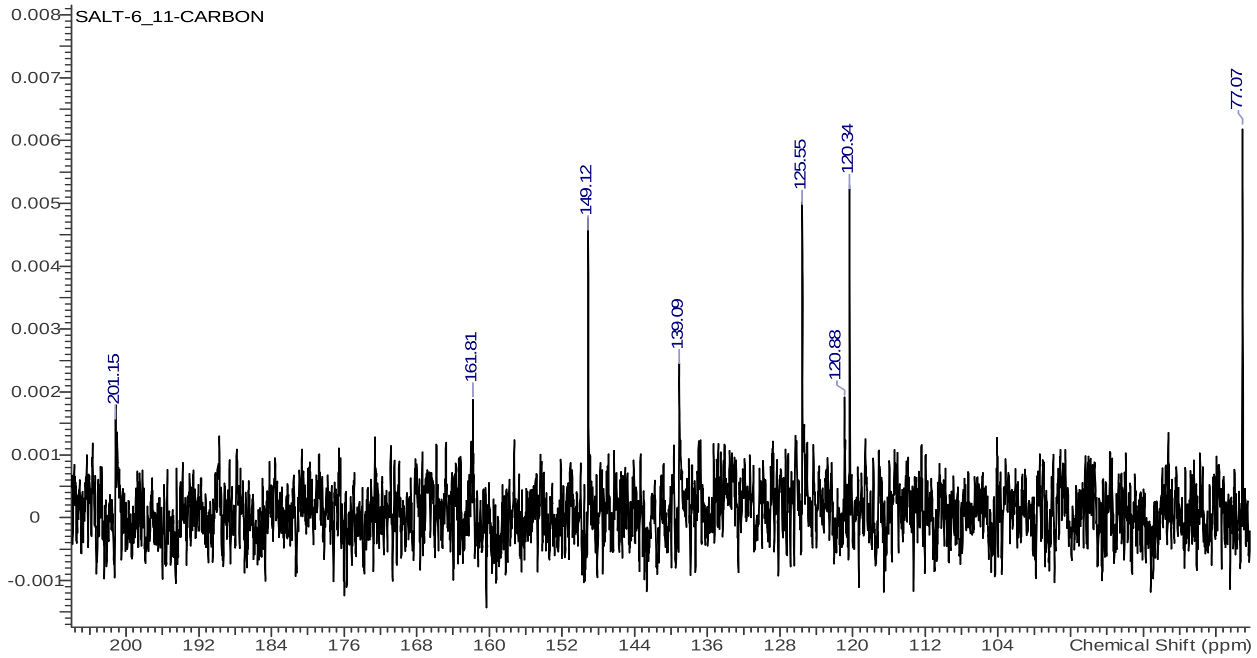

Figure S18. Zoomed-in region of Figure S16 from 76-205 ppm.

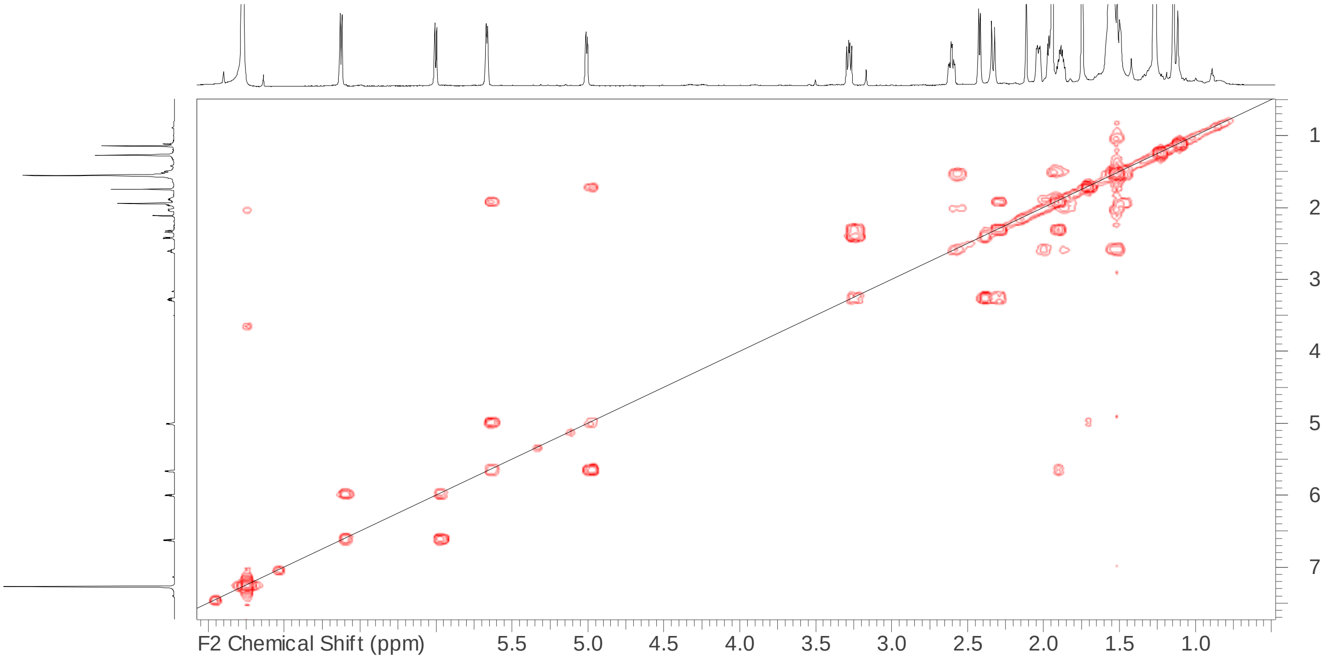

Figure S19. ${}^{1}H$-${}^{1}H$ COSY NMR spectrum for bathyptilone C (**3**) (500 MHz, $CDCl_3$).

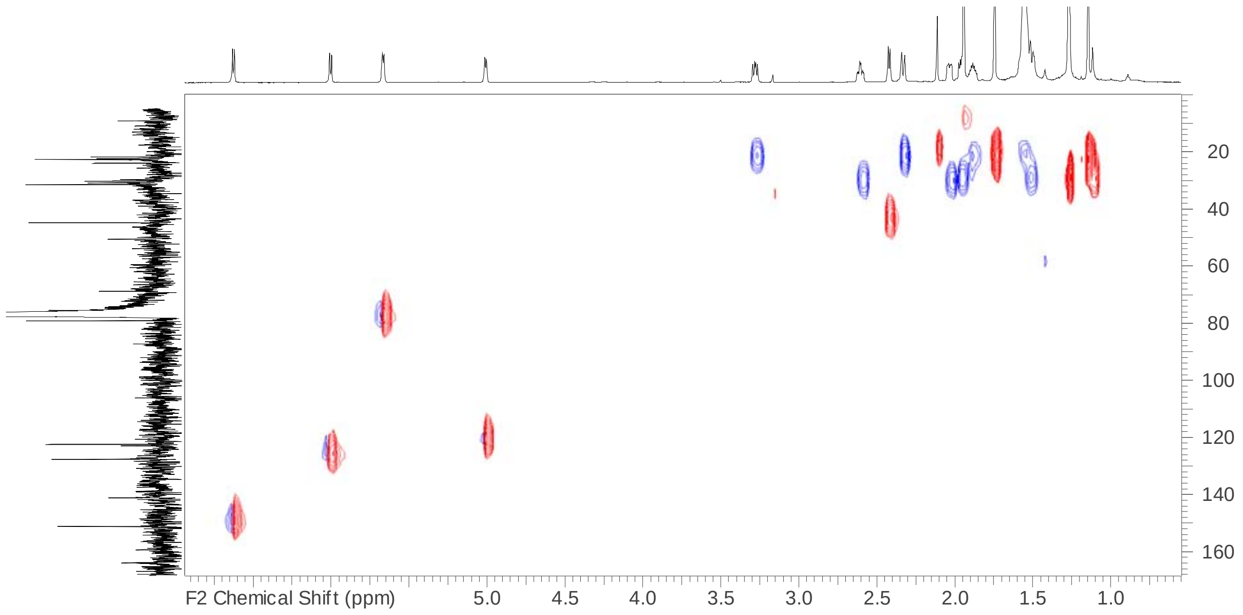

Figure S20. ^{1}H-^{13}C HSQC NMR spectrum for bathyptilone C (**3**) (800 MHz, $CDCl_3$).

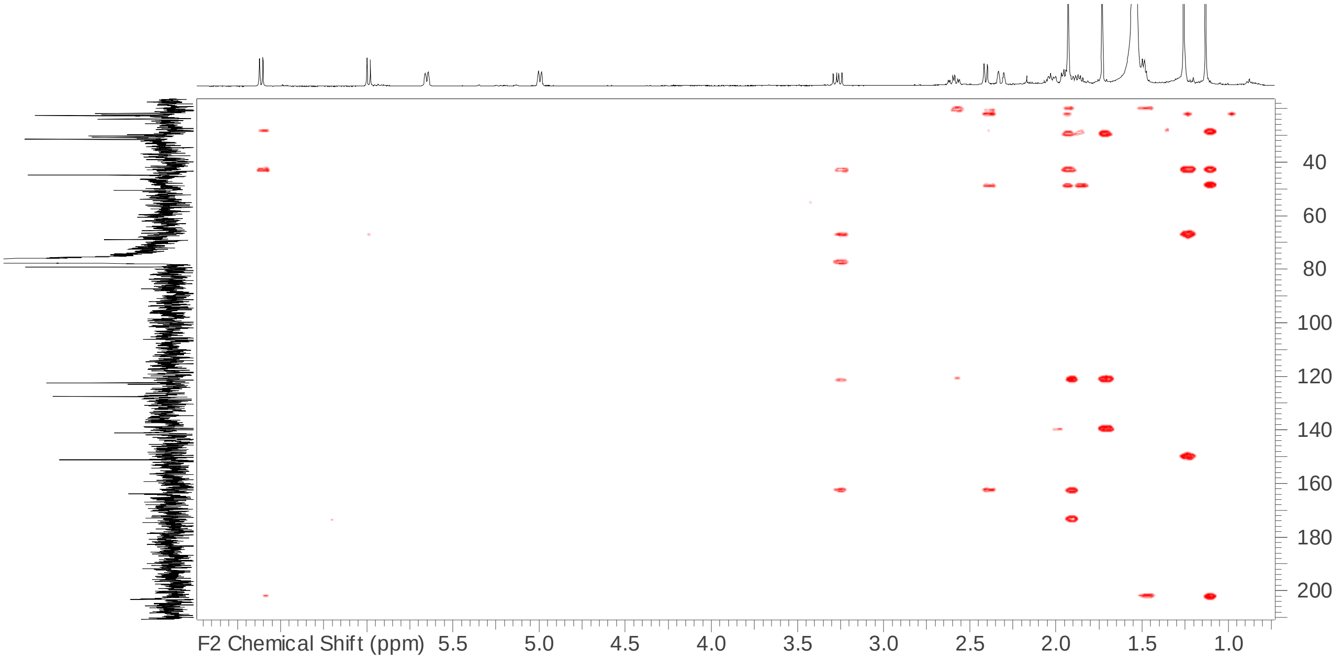

Figure S21. ^{1}H-^{13}C HMBC NMR spectrum for bathyptilone C (**3**) (500 MHz, $CDCl_3$).

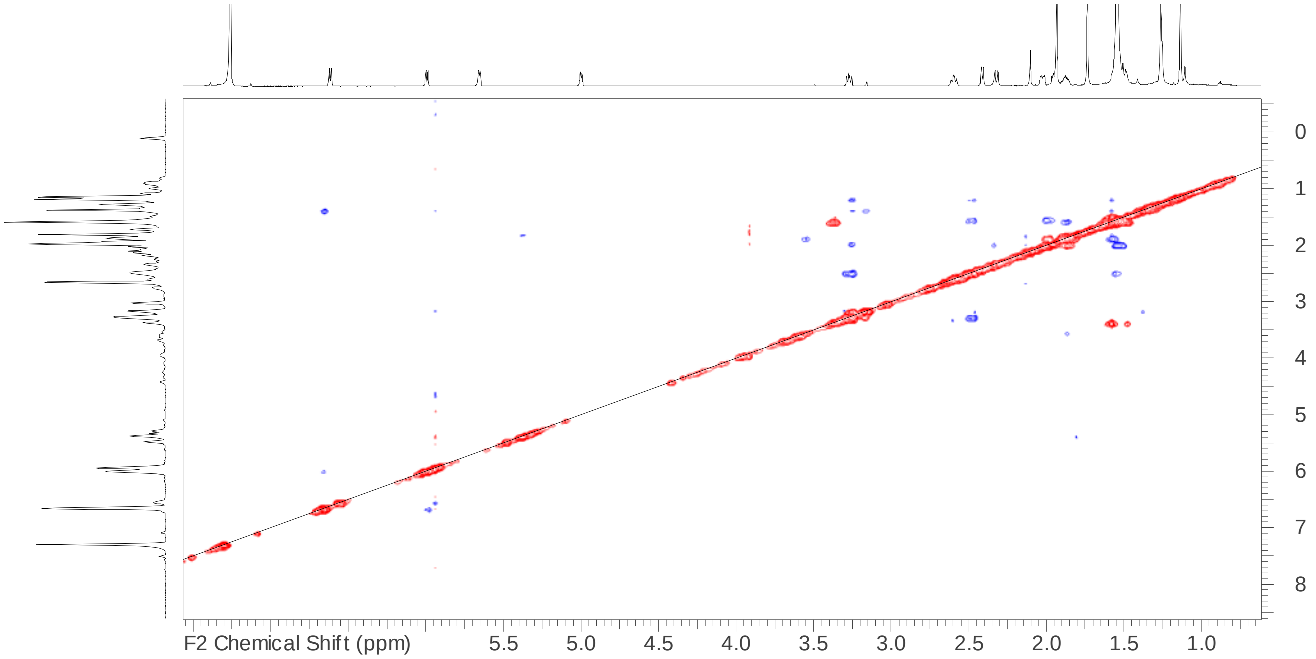

Figure S22. ^{1}H-^{1}H NOESY NMR spectrum for bathyptilone C (**3**) (800 MHz, $CDCl_3$).

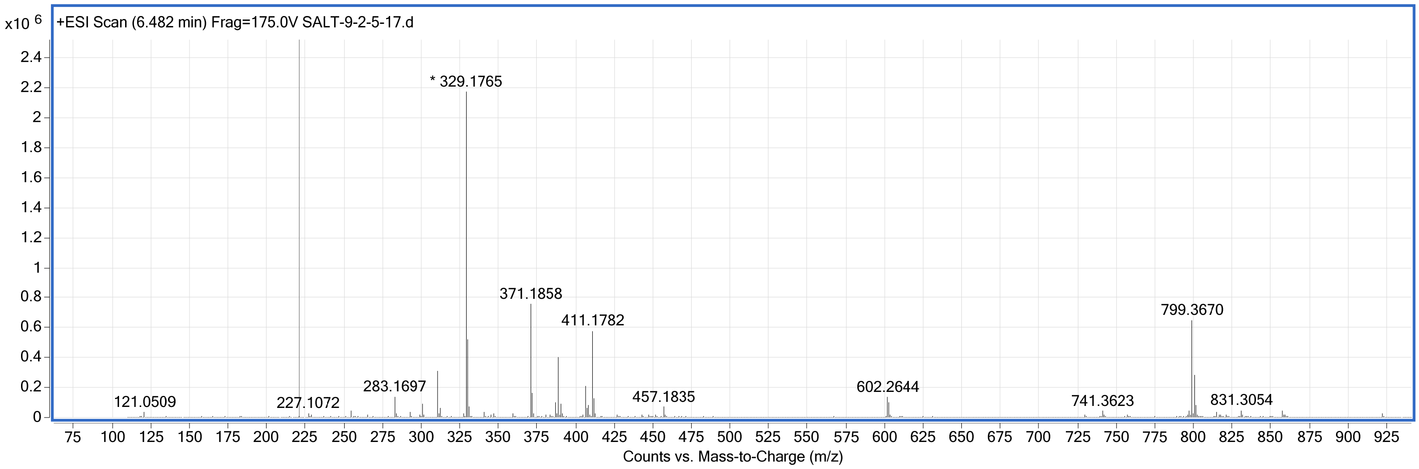

Figure S23. High resolution ESI-MS spectrum for bathyptilone C (**3**).

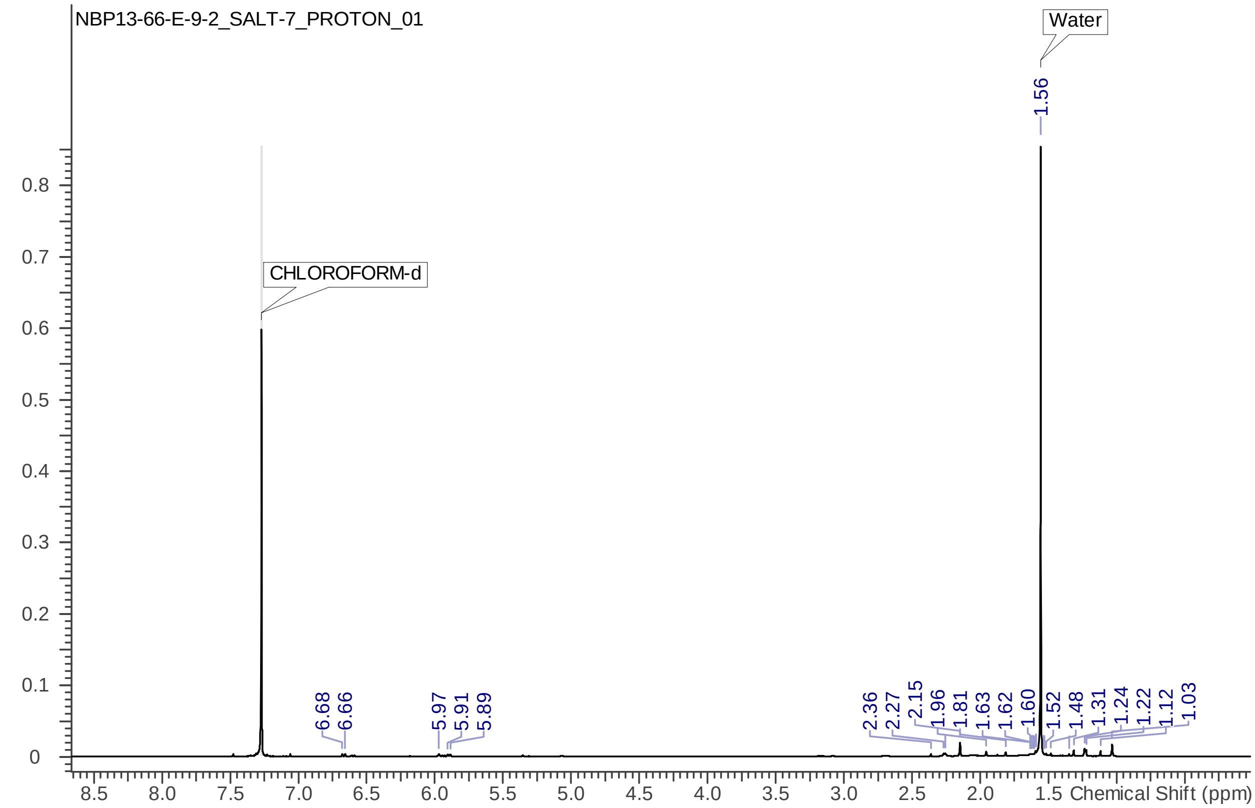

Figure S24. ^{1}H NMR spectrum for enbepeanone A (**4**) (500 MHz, $CDCl_3$).

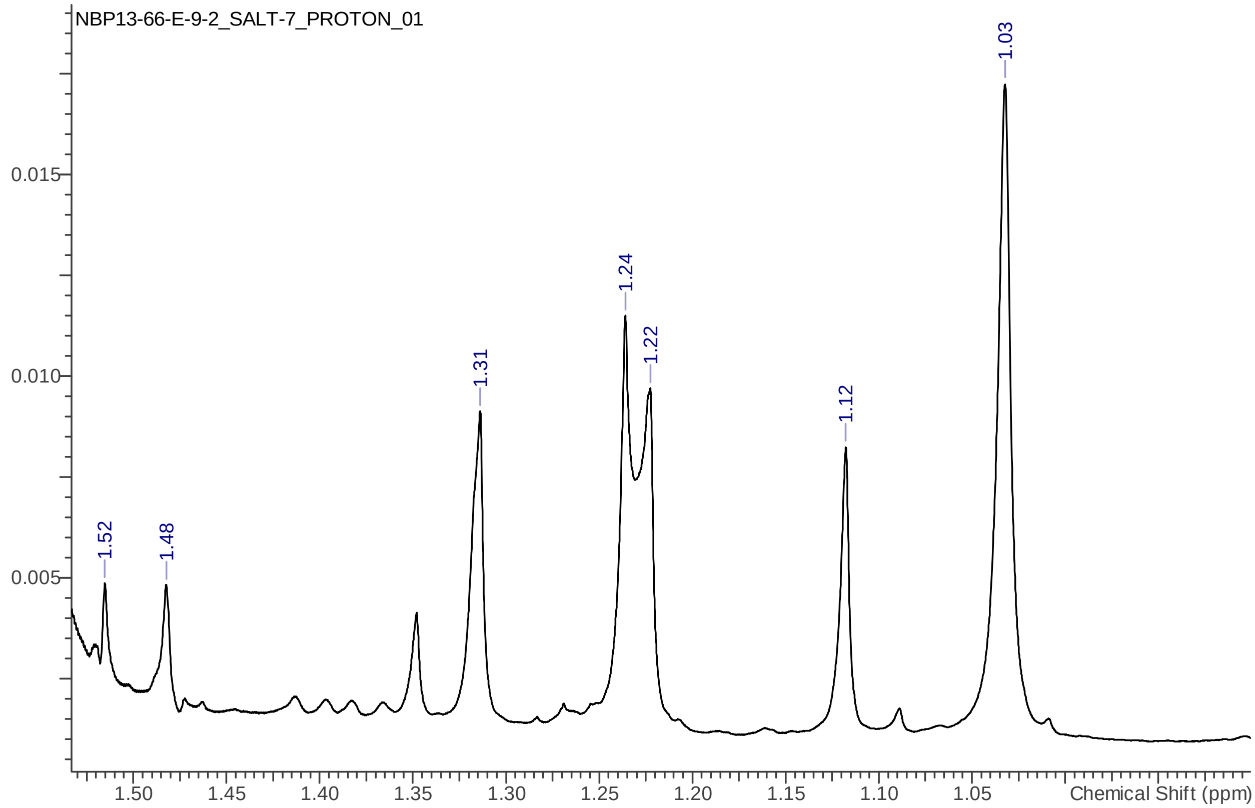

Figure S25. Zoomed-in region of Figure S24 from 0.9-1.53 ppm.

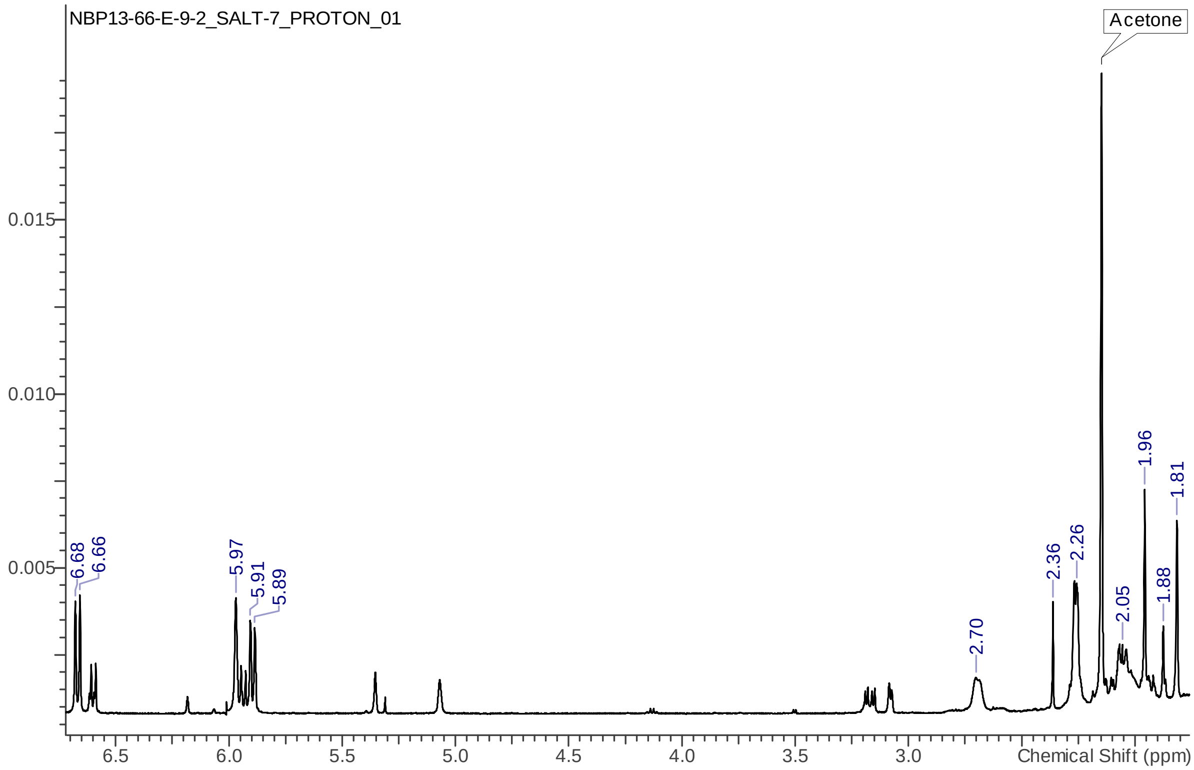

Figure S26. Zoomed-in region of Figure S24 from 1.59-6.7 ppm.

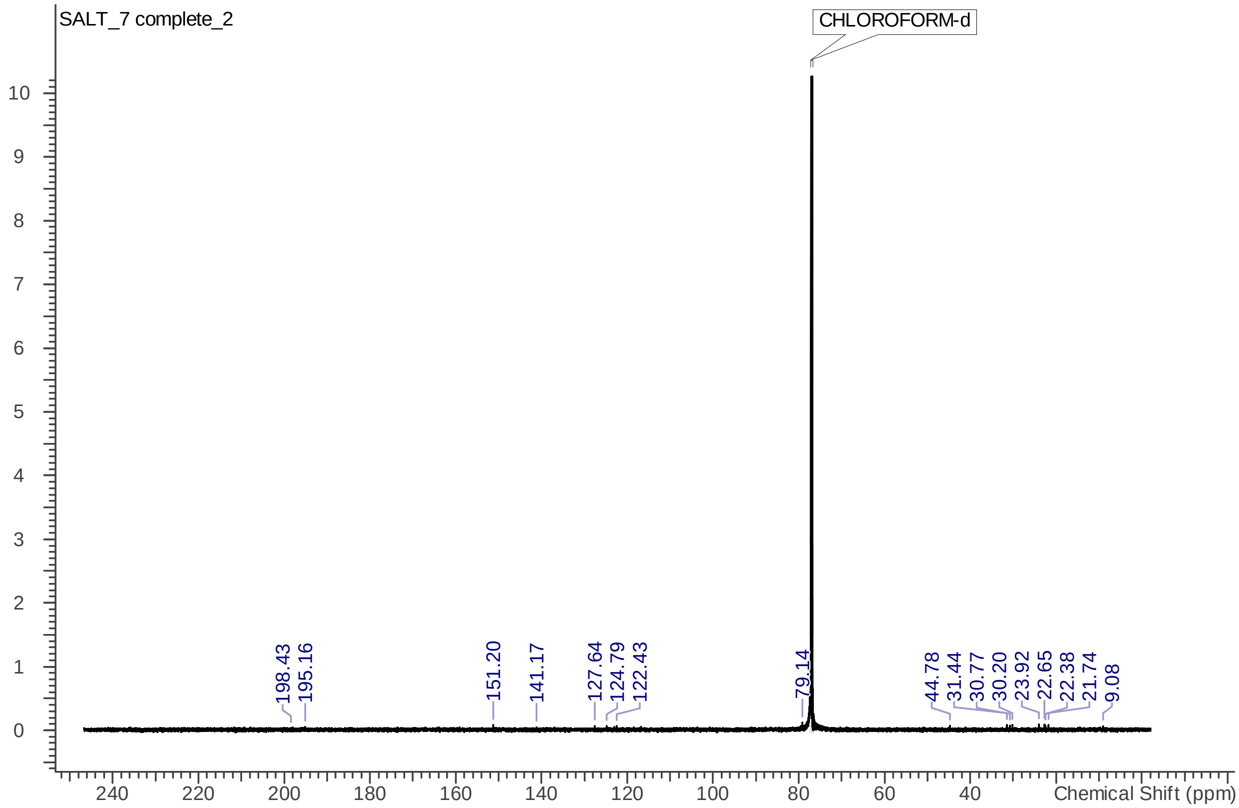

Figure S27. ^{13}C NMR spectrum for enbepeanone A (**4**) (200 MHz, $CDCl_3$).

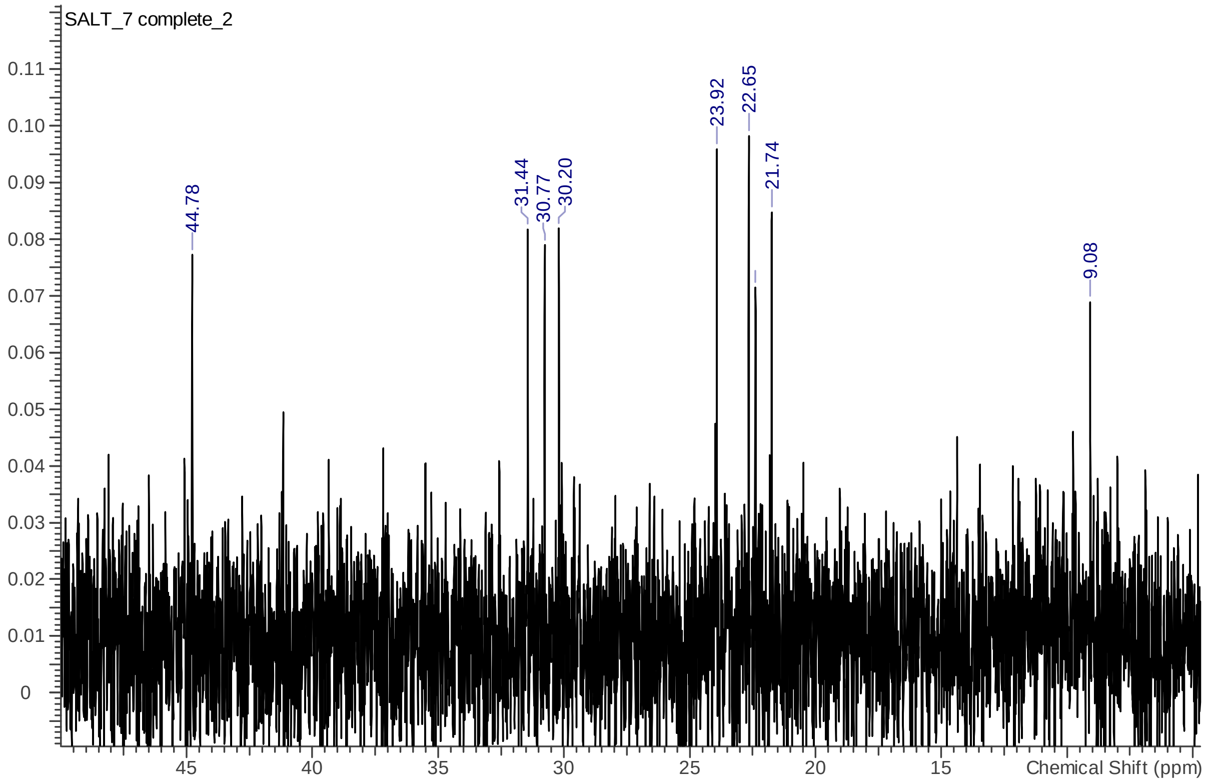

Figure S28. Zoomed in region of Figure S27 from 0-50 ppm.

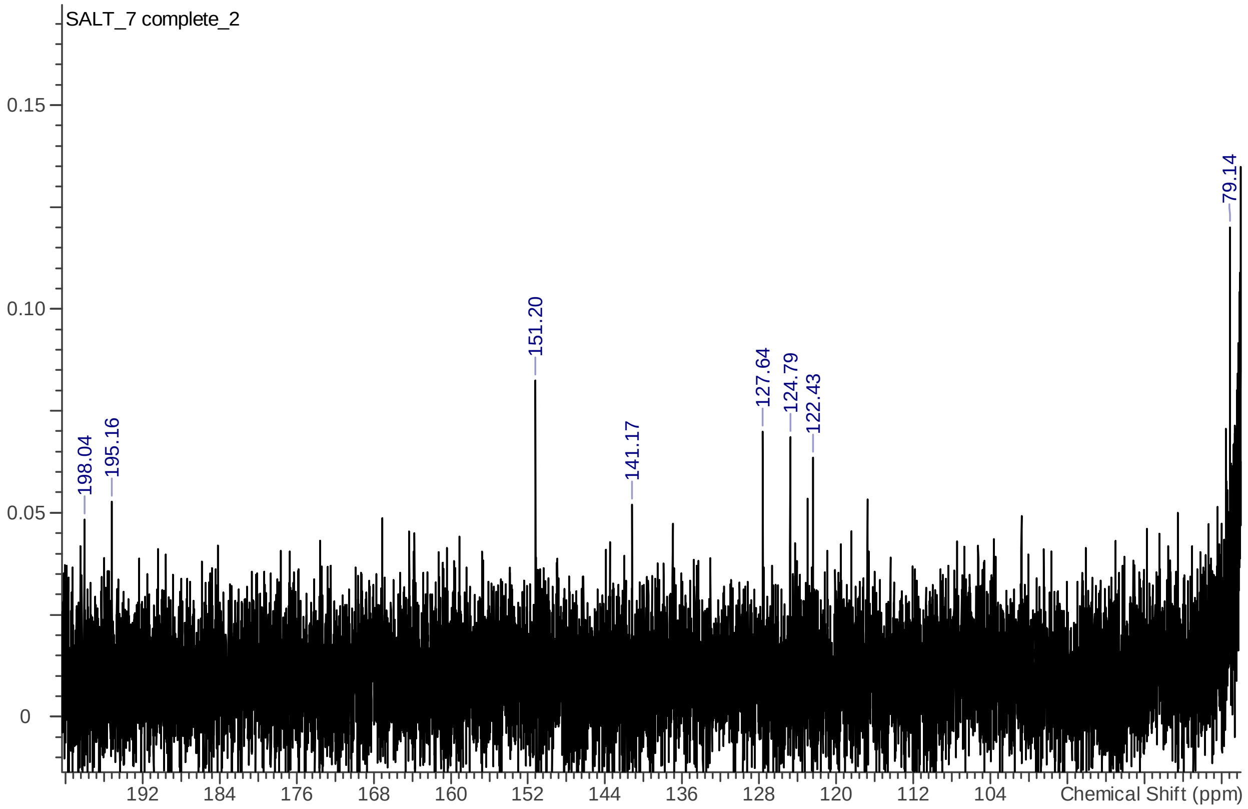

Figure S29. Zoomed in region of Figure S27 from 78-200 ppm.

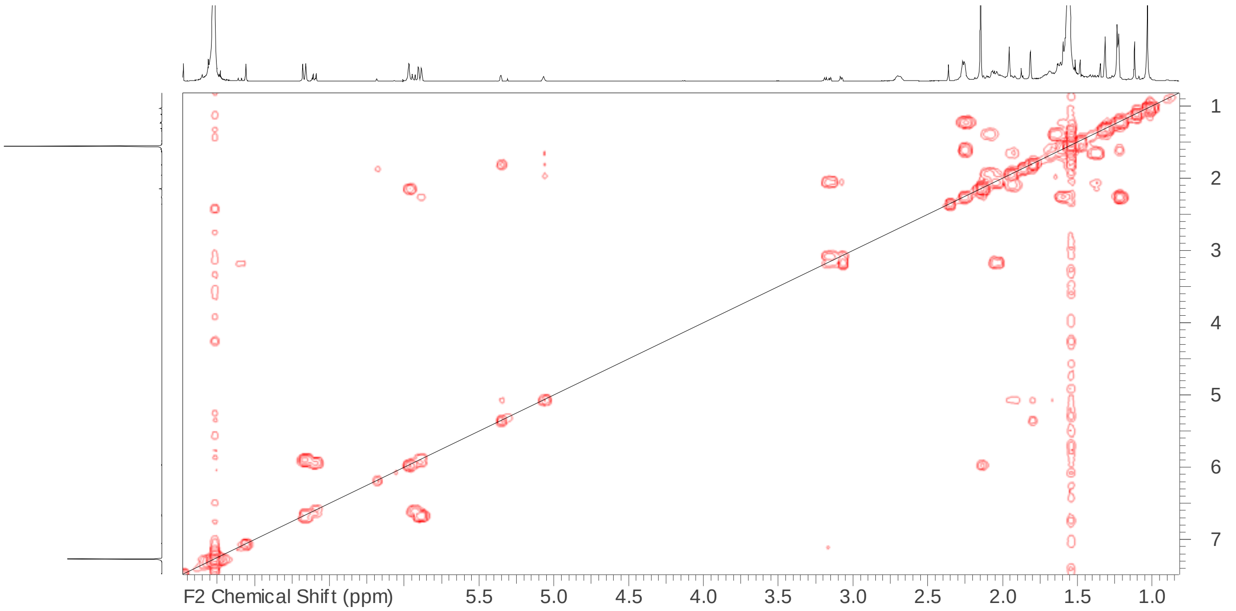

Figure S30. ^{1}H-^{1}H COSY NMR spectrum for enbepeanone A (**4**) (500 MHz, $CDCl_3$).

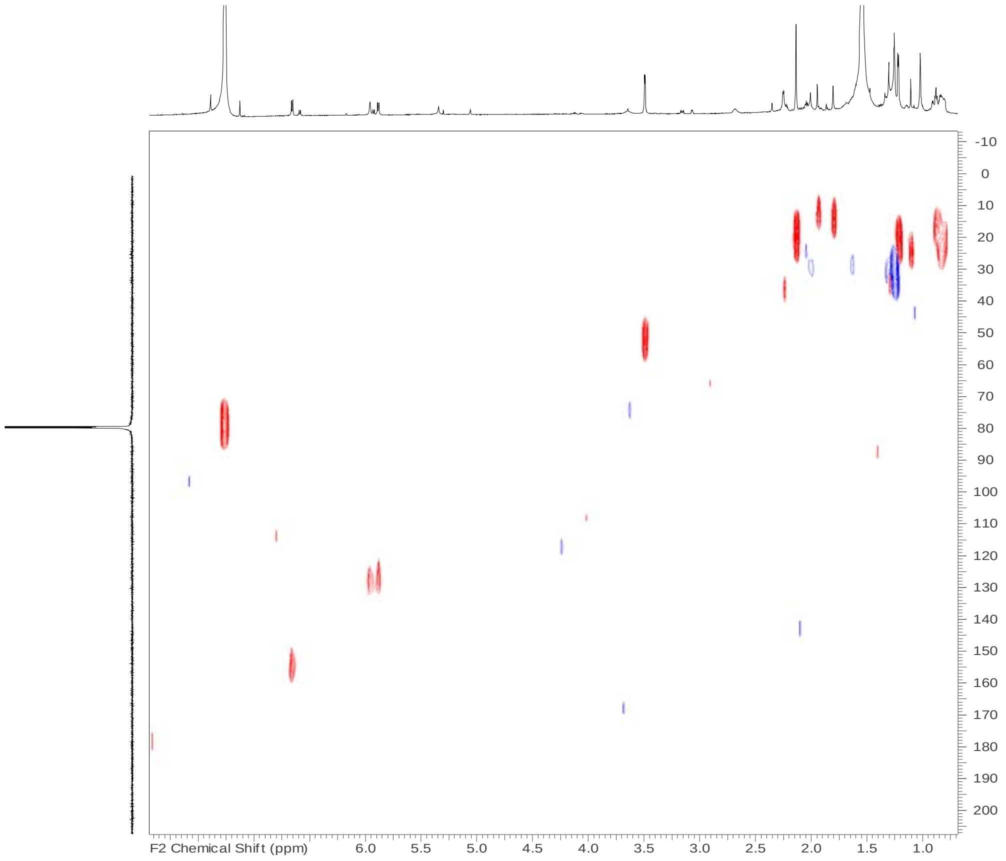

Figure S31. ^{1}H-^{13}C HSQC NMR spectrum for enbepeanone A (**4**) (800 MHz, $CDCl_3$).

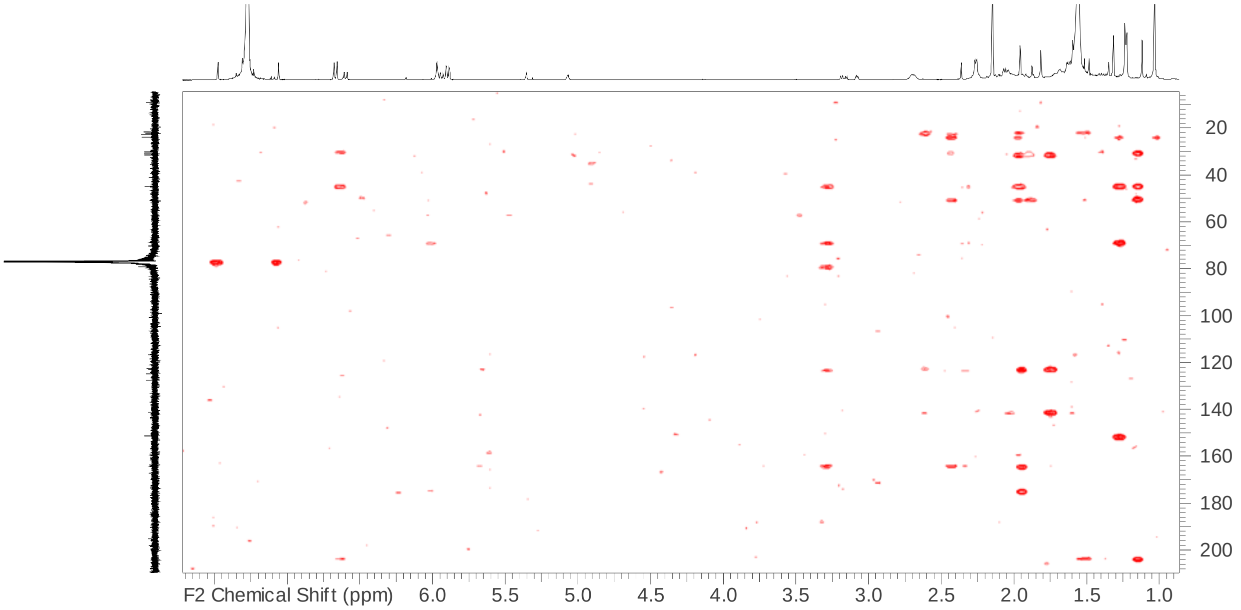

Figure S32. ^{1}H-^{13}C HMBC NMR spectrum for enbepeanone A (**4**) (500 MHz, $CDCl_3$).

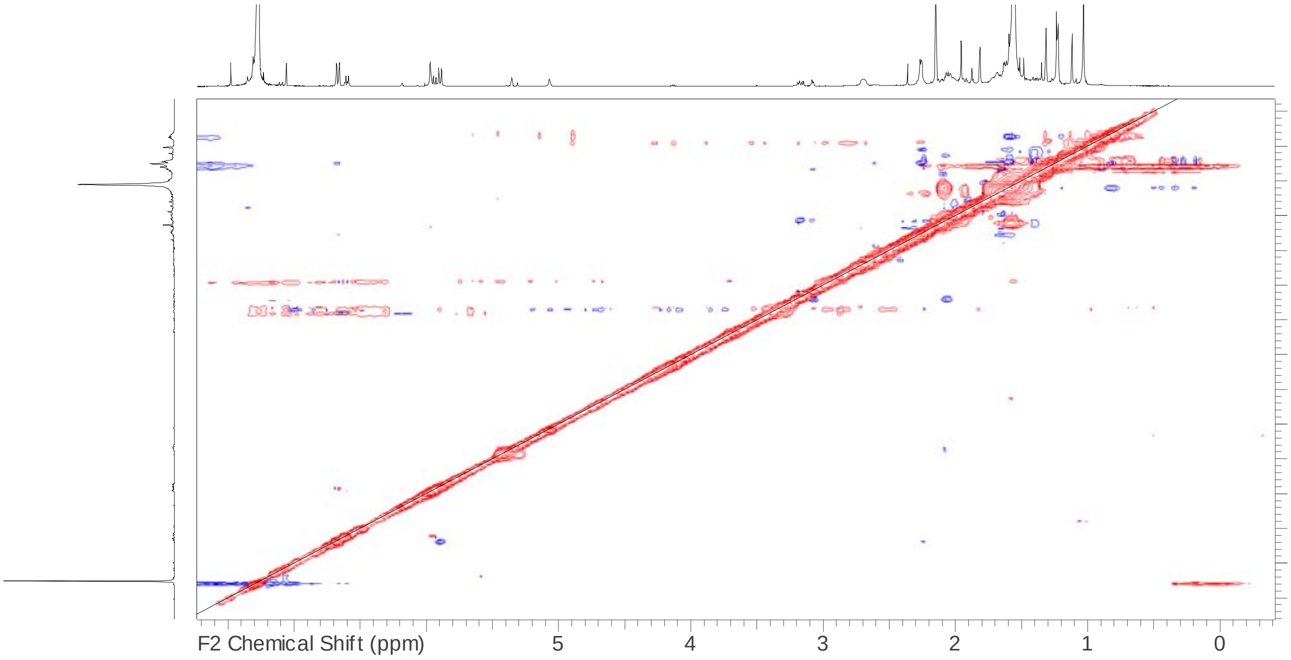

Figure S33. ^{1}H-^{1}H NOESY NMR spectrum for enbepeanone A (**4**) (500 MHz, $CDCl_3$).

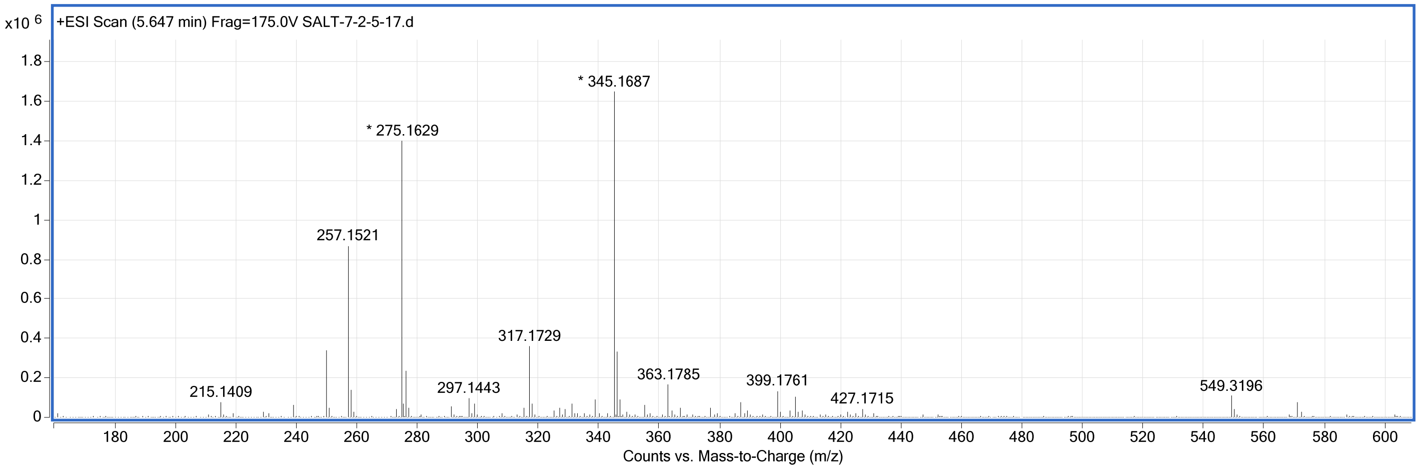

Figure S34. High resolution ESI-MS spectrum for enbepeanone A (**4**).

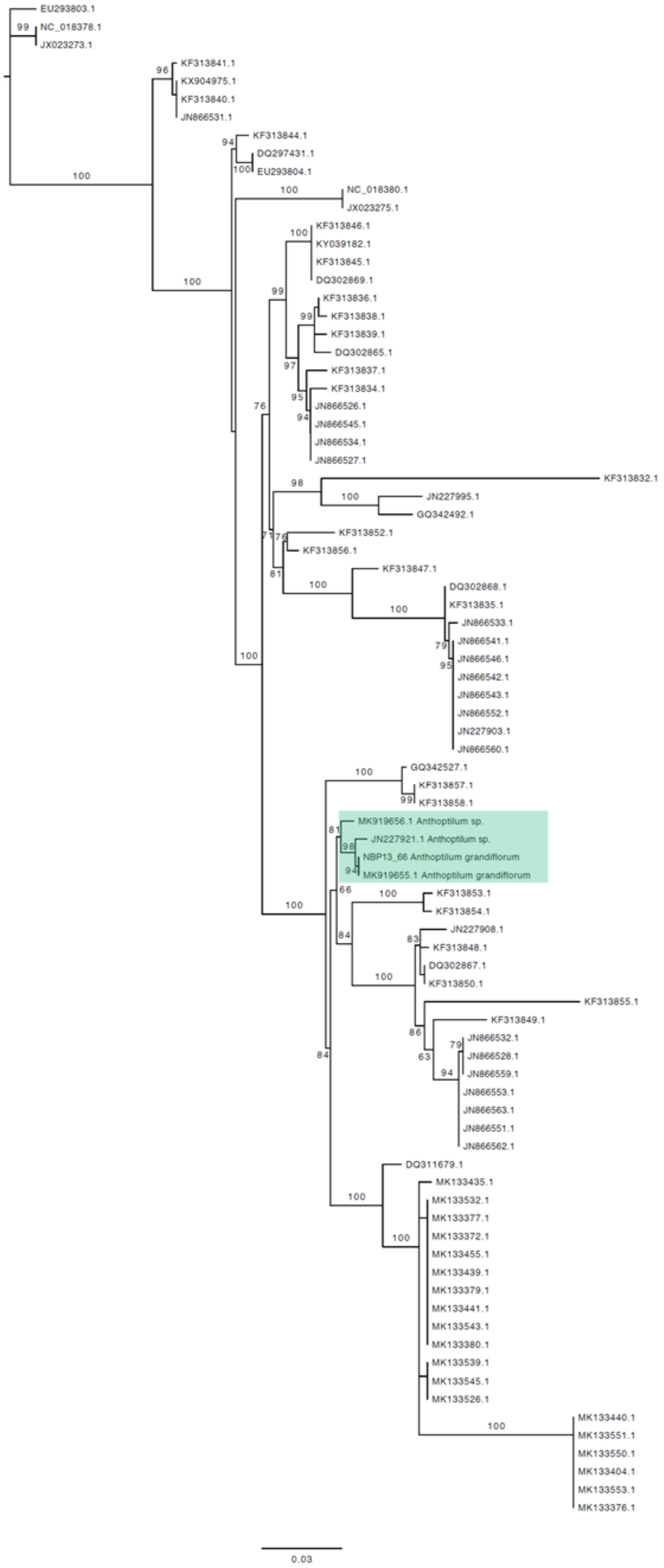

Figure S35. Maximum Likelihood tree topology comparing our Anthoptilum msh1 sequences with those available on Genbank

Table S1. Crystal data and structure refinement for bathyptilone A (1).	
Identification code	NBP13_66_E
Empirical formula	$C_{20}H_{26}O_5$
Formula weight	346.41
Temperature/K	100.01
Crystal system	orthorhombic
Space group	$P2_12_12_1$
a/Å	8.7926(2)
b/Å	12.2611(2)
c/Å	16.4222(3)
α/°	90
β/°	90
γ/°	90
Volume/Å^3	1770.43(6)
Z	4
ρ_{calc}g/cm^3	1.300
μ/mm^{-1}	0.753
F(000)	744.0
Crystal size/mm^3	0.093 × 0.038 × 0.034
Radiation	CuKα (λ = 1.54178)
2Θ range for data collection/°	9 to 132.984
Index ranges	-10 ≤ h ≤ 10, -14 ≤ k ≤ 14, -19 ≤ l ≤ 19
Reflections collected	23978
Independent reflections	3123 [R_{int} = 0.0453, R_{sigma} = 0.0213]
Data/restraints/parameters	3123/0/238
Goodness-of-fit on F^2	1.088
Final R indexes [I>=2σ (I)]	R_1 = 0.0288, wR_2 = 0.0674
Final R indexes [all data]	R_1 = 0.0312, wR_2 = 0.0688
Largest diff. peak/hole / e Å^{-3}	0.19/-0.18
Flack parameter	-0.04(6)

Table S2. Crystal data and structure refinement for bathyptilone B (2).	
Identification code	NB_P13_66_E_4_2
Empirical formula	$C_{22}H_{28}O_6$
Formula weight	388.44
Temperature/K	100.02
Crystal system	Monoclinic
Space group	$P2_1$
a/Å	9.5139(4)
b/Å	10.3255(5)
c/Å	10.4615(4)
α/°	90
β/°	90.924(3)
γ/°	90
Volume/Å^3	1027.56(8)
Z	2
ρ_{calc}g/cm^3	1.255
μ/mm^{-1}	0.744
F(000)	416.0
Crystal size/mm^3	0.04 × 0.02 × 0.005
Radiation	CuKα (λ = 1.54178)
2Θ range for data collection/°	8.452 to 154.192
Index ranges	-11 ≤ h ≤ 11, -13 ≤ k ≤ 13, -12 ≤ l ≤ 13
Reflections collected	8460
Independent reflections	3614 [R_{int} = 0.1118, R_{sigma} = 0.1052]
Data/restraints/parameters	3614/1/262
Goodness-of-fit on F^2	1.041
Final R indexes [I>=2σ (I)]	R_1 = 0.0576, wR_2 = 0.1076
Final R indexes [all data]	R_1 = 0.0859, wR_2 = 0.1184
Largest diff. peak/hole / e Å^{-3}	0.25/-0.24
Flack parameter	0.2(3)

**Table S3. Crystal data and structure refinement for bathyptilone C (3).**	
Identification code	ST_NBP13_66_E_9_4_b
Empirical formula	$C_{20}H_{26}O_4$
Formula weight	330.41
Temperature/K	99.99
Crystal system	orthorhombic
Space group	$P2_12_12_1$
a/Å	8.2529(2)
b/Å	10.8806(3)
c/Å	19.0878(5)
α/°	90
β/°	90
γ/°	90
Volume/Å^3	1714.02(8)
Z	4
ρ_{calc}g/cm^3	1.280
μ/mm^{-1}	0.707
F(000)	712.0
Crystal size/mm^3	0.467 × 0.058 × 0.055
Radiation	CuKα (λ = 1.54178)
2Θ range for data collection/°	9.266 to 154.388
Index ranges	-10 ≤ h ≤ 9, -13 ≤ k ≤ 13, -23 ≤ l ≤ 23
Reflections collected	25817
Independent reflections	3597 [R_{int} = 0.0725, R_{sigma} = 0.0359]
Data/restraints/parameters	3597/0/225
Goodness-of-fit on F^2	1.071
Final R indexes [I>=2σ (I)]	R_1 = 0.0356, wR_2 = 0.0852
Final R indexes [all data]	R_1 = 0.0398, wR_2 = 0.0885
Largest diff. peak/hole / e Å^{-3}	0.27/-0.18
Flack parameter	0.03(9)

**Table S4. Crystal data and structure refinement for enbepeanone A (4).**	
Identification code	NB_P13_66_E_9_2_Salt_7
Empirical formula	$C_{17}H_{22}O_3$
Formula weight	274.34
Temperature/K	100.0
Crystal system	orthorhombic
Space group	$P2_12_12_1$
a/Å	7.1483(2)
b/Å	7.7391(2)
c/Å	25.4330(7)
α/°	90
β/°	90
γ/°	90
Volume/Å^3	1406.99(7)
Z	4
ρ_{calc}g/cm^3	1.295
μ/mm^{-1}	0.698
F(000)	592.0
Crystal size/mm^3	0.2 × 0.06 × 0.02
Radiation	CuKα (λ = 1.54178)
2Θ range for data collection/°	6.95 to 153.75
Index ranges	-8 ≤ h ≤ 9, -9 ≤ k ≤ 9, -31 ≤ l ≤ 31
Reflections collected	9932
Independent reflections	2884 [R_{int} = 0.0551, R_{sigma} = 0.0432]
Data/restraints/parameters	2884/0/188
Goodness-of-fit on F^2	1.105
Final R indexes [I>=2σ (I)]	R_1 = 0.0374, wR_2 = 0.0789
Final R indexes [all data]	R_1 = 0.0455, wR_2 = 0.0825
Largest diff. peak/hole / e Å^{-3}	0.19/-0.23
Flack parameter	-0.03(13)

**Table S5**. Bijvoet-Pair analysis and Bayesian statistics	
**bathyptilone A (1)**	**Enbepeanone A (4)**
Space Group P212121<br>Wavelength 1.54178<br>Flack x .... -0.04(6)<br>Parsons z .. -0.03(7)<br><br><br>Bijvoet Pairs 1319<br>Coverage ... 100<br>DiffCalcMax. 40.69<br>Outlier Crit 81.38<br>Scatter Plot<br>Sigma Crit.. 0.25<br>Select Pairs 270<br>Number Plus 176<br>Number Minus 94<br>Slope ...... 1.139<br><br><br>Student-T Prob. Plot<br>Sample Size. 1309<br>Corr. Coeff. 0.999<br>Intercept .. 0.008<br>Slope ...... 0.856<br><br><br>Bayesian Statistics<br>Student_T Nu 100<br>Select Pairs 1319<br>Theta_Min .. 6.76<br>Theta_Max .. 66.49<br>P2(true).... 1.000<br>P3(true).... 1.000<br>P3(rac-twin) 0.9E-18<br>P3(false) .. 0.1E-67<br>G .... 1.0641<br>G (su) ..... 0.1164<br>Hooft y ... -0.03(6)	Space Group P212121<br>Wavelength 1.54178<br>Flack x .... -0.03(13)<br>Parsons z .. 0.03(14)<br><br><br>Bijvoet Pairs 1162<br>Coverage ... 94<br>DiffCalcMax. 23.94<br>Outlier Crit 47.88<br>Scatter Plot<br>Sigma Crit.. 0.25<br>Select Pairs 44<br>Number Plus 32<br>Number Minus 12<br>Slope ...... 0.860<br><br><br>Student-T Prob. Plot<br>Sample Size. 1152<br>Corr. Coeff. 0.999<br>Intercept .. 0.003<br>Slope ...... 0.851<br><br><br>Bayesian Statistics<br>Student_T Nu 100<br>Select Pairs 1162<br>Theta_Min .. 8.62<br>Theta_Max .. 76.45<br>P2(true).... 1.000<br>P3(true).... 1.000<br>P3(rac-twin) 0.4E-03<br>P3(false) .. 0.4E-14<br>G .... 0.9433<br>G (su) ..... 0.2388<br>Hooft y ... 0.03(12)
